# Supplementary material for: Genome-wide association meta-analysis identifies risk loci for abdominal aortic aneurysm and highlights PCSK9 as a therapeutic target
Source: Nat Genet. 2023 Oct 16;55(11):1831–42. doi: 10.1038/s41588-023-01510-y (PMC10632148; doi:10.1038/s41588-023-01510-y)
Supplement: Supplementary file 1 — Supplementary Figs. 1–17, Supplementary Note, cohort acknowledgments and consortium authors. [file 41588_2023_1510_MOESM1_ESM.pdf]

nature genetics

Article

https://doi.org/10.1038/s41588-023-01510-y

Genome-wide association meta-analysis identifies risk loci for abdominal aortic aneurysm and highlights PCSK9 as a therapeutic target

In the format provided by the authors and unedited

Table of Contents

Supplementary Figures (1-17) ……..…………………………………...……………………..2

Supplementary Notes (Results and Methods) ……………………………………………...20

References ………………………………………………………………………………….35

Cohort acknowledgements …………………………………………………………………38

Consortium authors ………………………………………………………………………...39

Supplementary figures

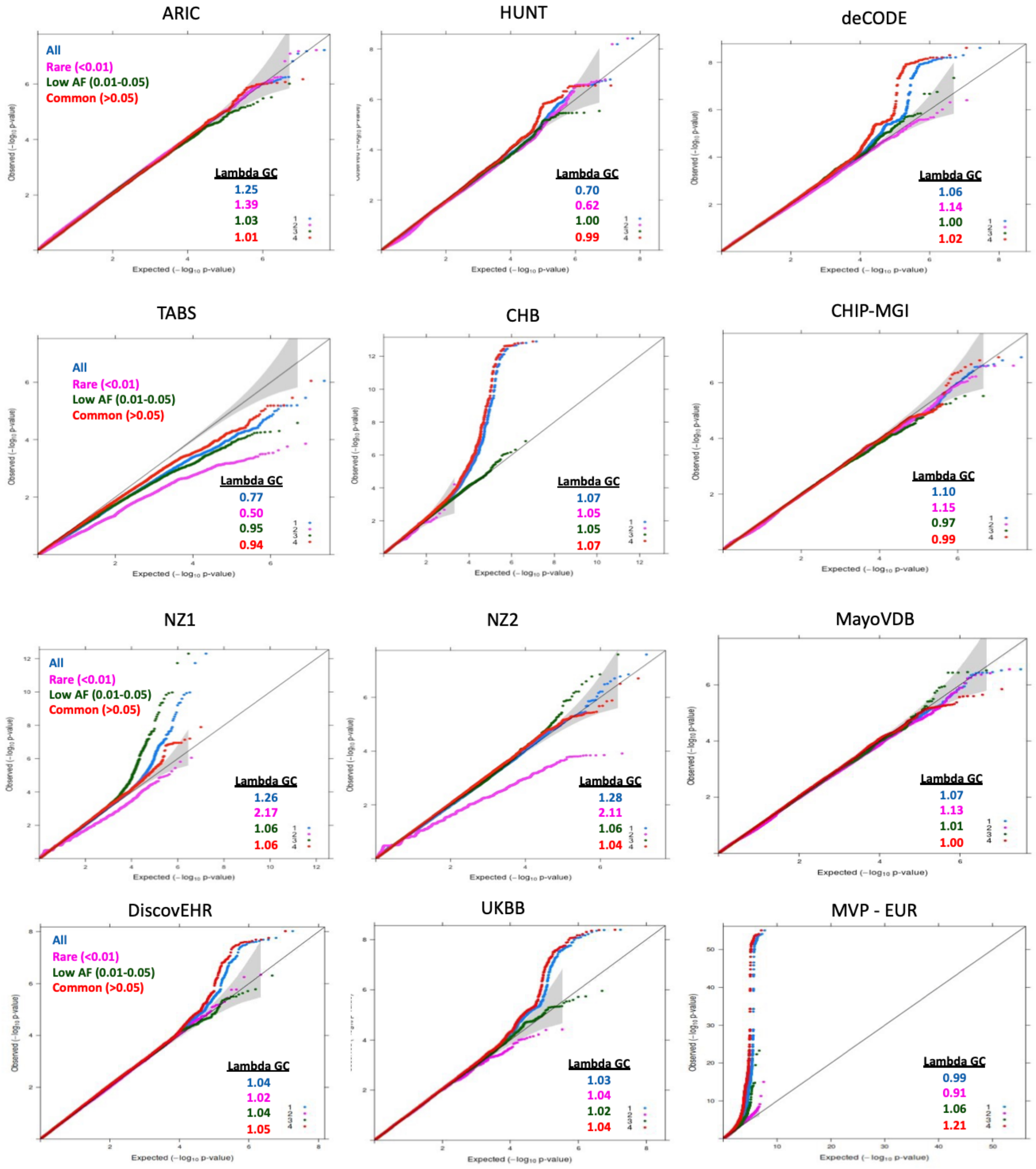

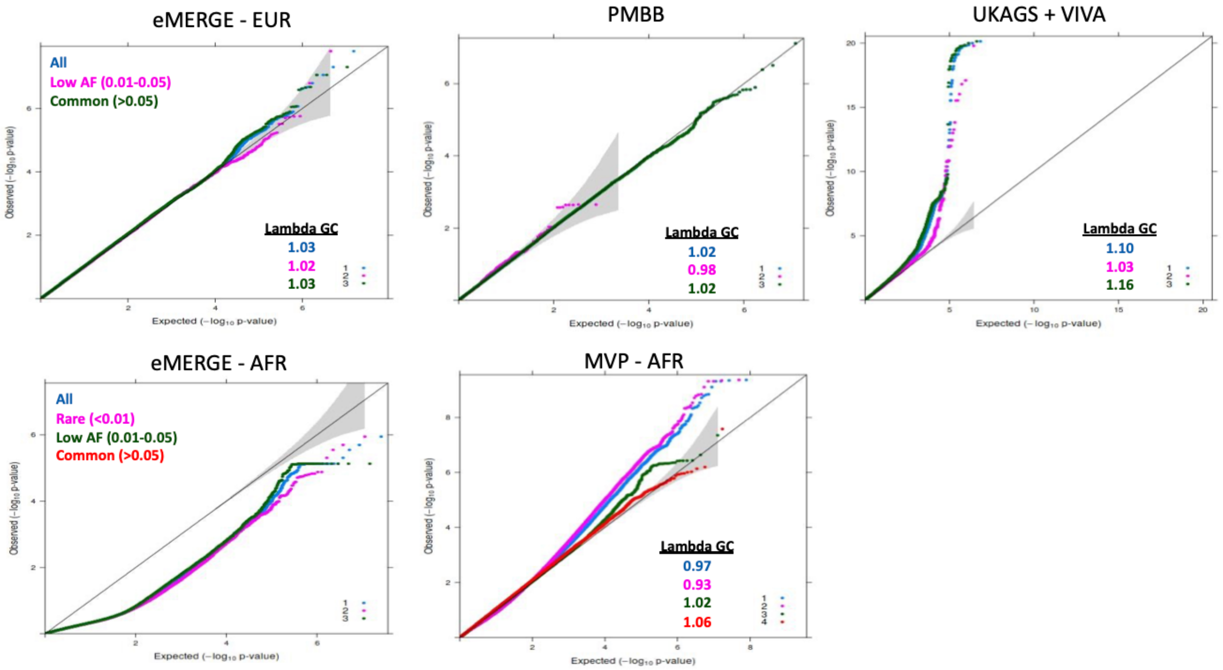

Supplementary Figure 1: QQ plots of 17 AAA GWAS summary statistics from 14 discovery cohorts. The expected logistic regression association p-values versus the observed p-values for AAA association are displayed. Sample sizes and analytical methods in discovery cohorts are listed in **Supplementary Table 1.** All p-values are two-sided. The confidence intervals were calculated with assumption that standard uniform order statistics follow a beta distribution (Default settings in https://genome.sph.umich.edu/wiki/Code_Sample:_Generating_QQ_Plots_in_R).

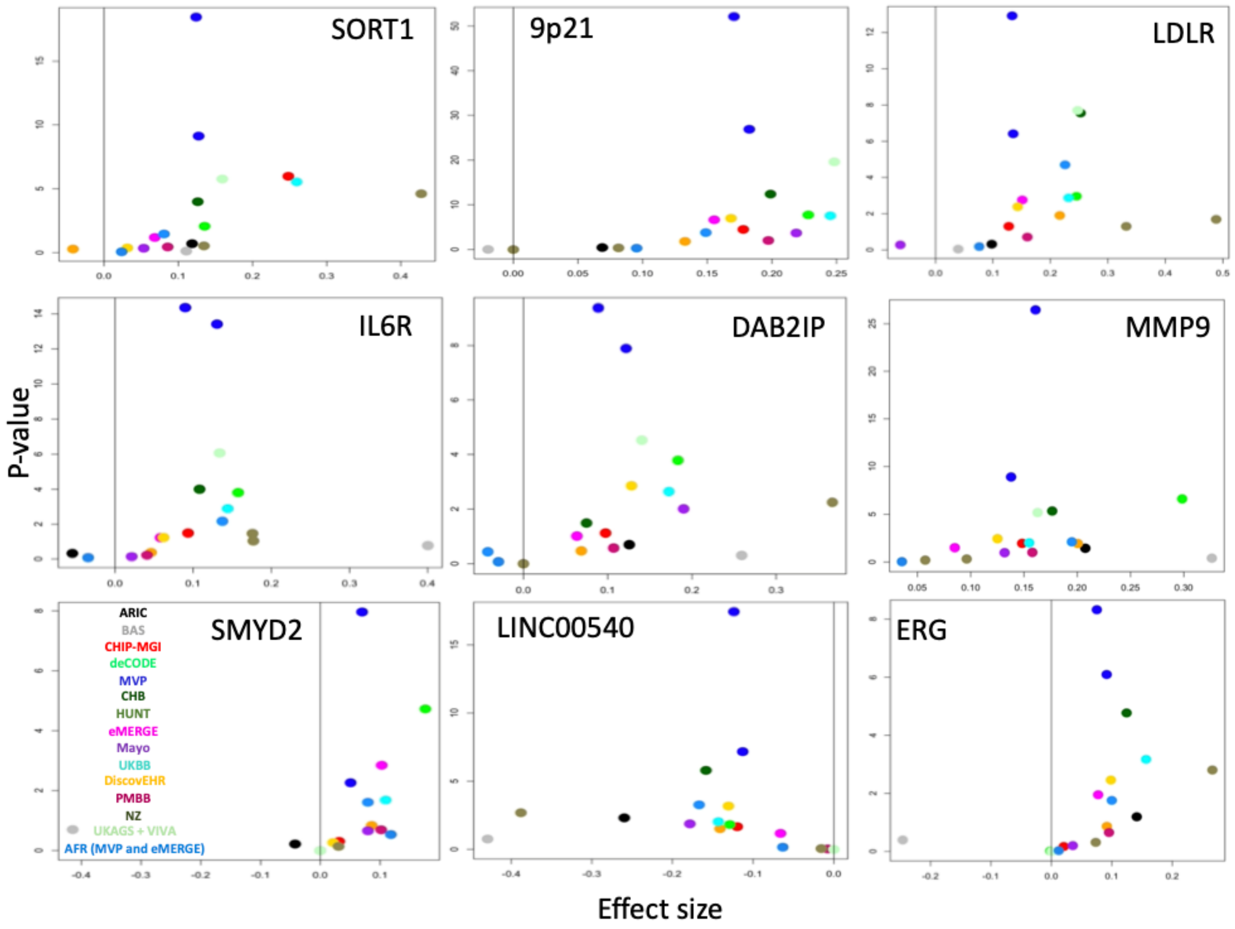

Supplementary Figure 2: Plot of GWAS effect size estimation (X-axis) against -$\log_{10}$p-value (Y-axis) for 9 known index variants of AAA in discovery cohorts. Sample sizes and analytical methods in discovery cohorts are listed in **Supplementary Table 1.** All p-values are two-sided.

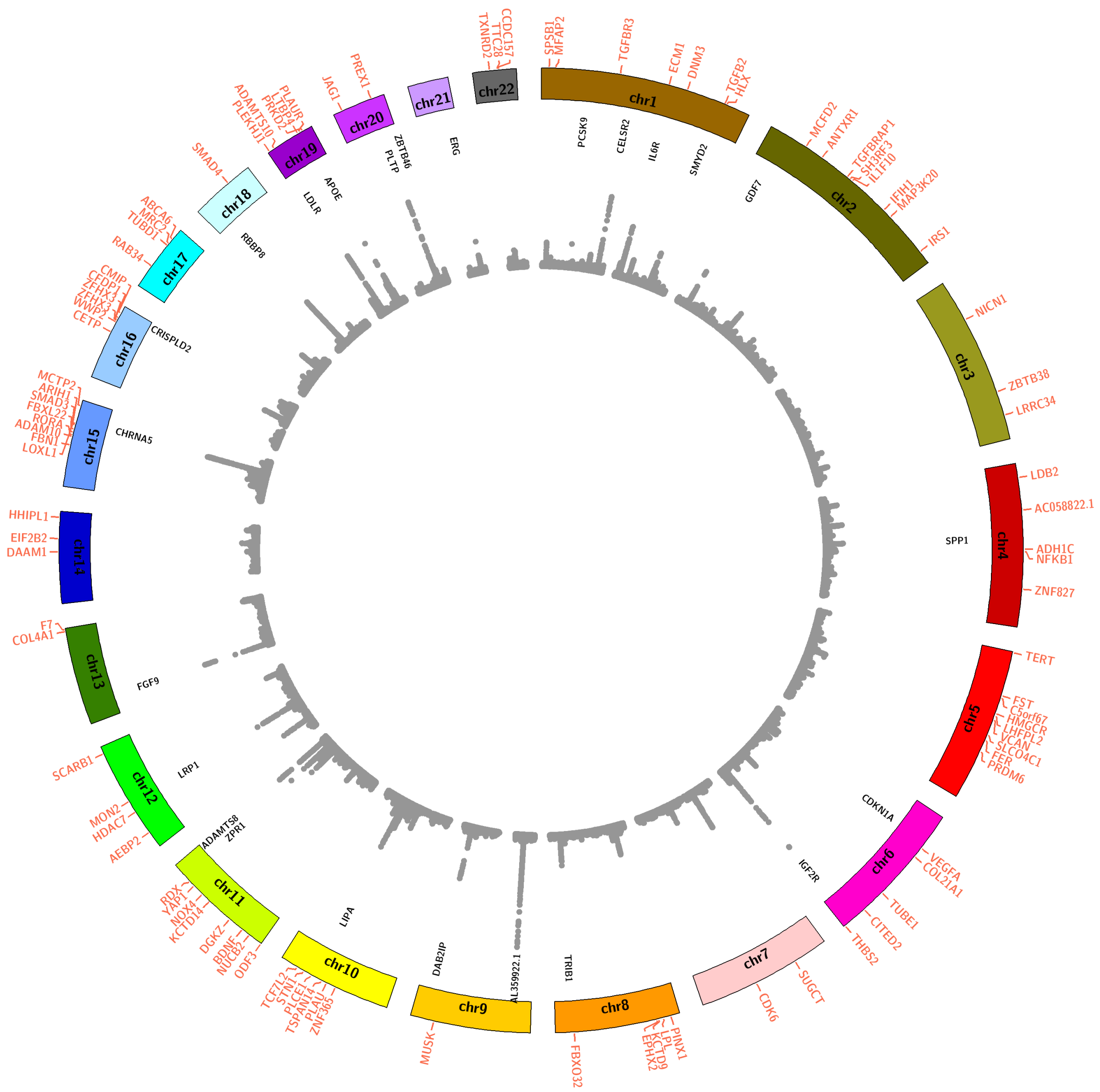

Supplementary Figure 3: Manhattan plot of AAAgen GWAS meta-analysis (N_{case}=39,221; $N_{control}$=1,086,107). Meta-analysis of 17 GWAS summary statistics from 14 discovery cohorts was performed by METAL[1] in standard error mode with genomic control correction. Novel loci are in red font and known loci are in black font. Variants with $P > 10^{-3}$ and $P < 10^{-60}$ (9p21) were excluded for better visualization. All p-values are two-sided.

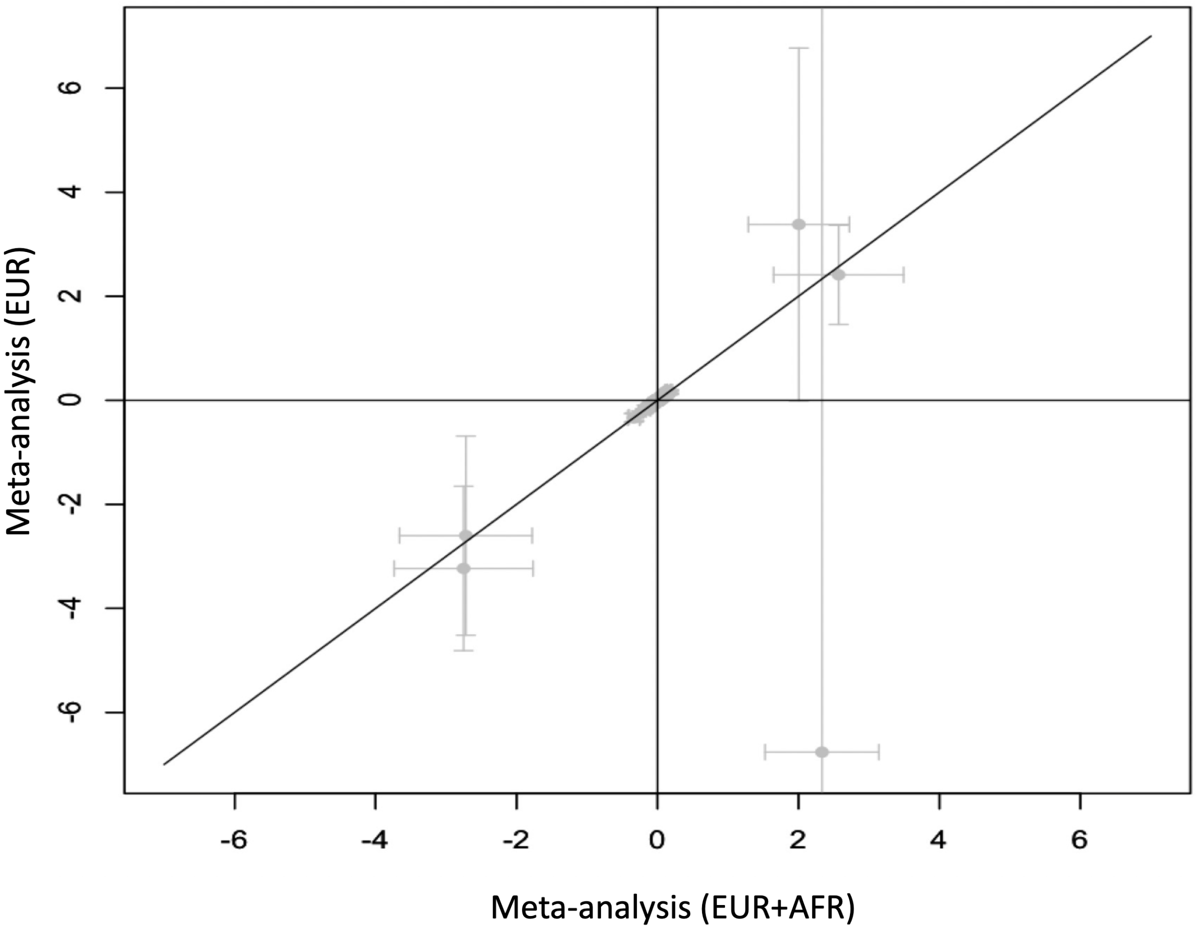

Supplementary Figure 4: Comparison of index variant effect estimates (95% CI) between AAAgen GWAS meta-analysis with (X-axis; N_{case}=39,221; $N_{control}$=1,086,107) or without AFR ancestry (Y-axis; N_{case}=37,214; $N_{control}$=997,776) summary statistics. Consistent effect estimates were observed for index variant with MAF > 0.01. Five rare index variants (off-diagonal) were excluded from the follow-up analyses after sensitivity analysis.

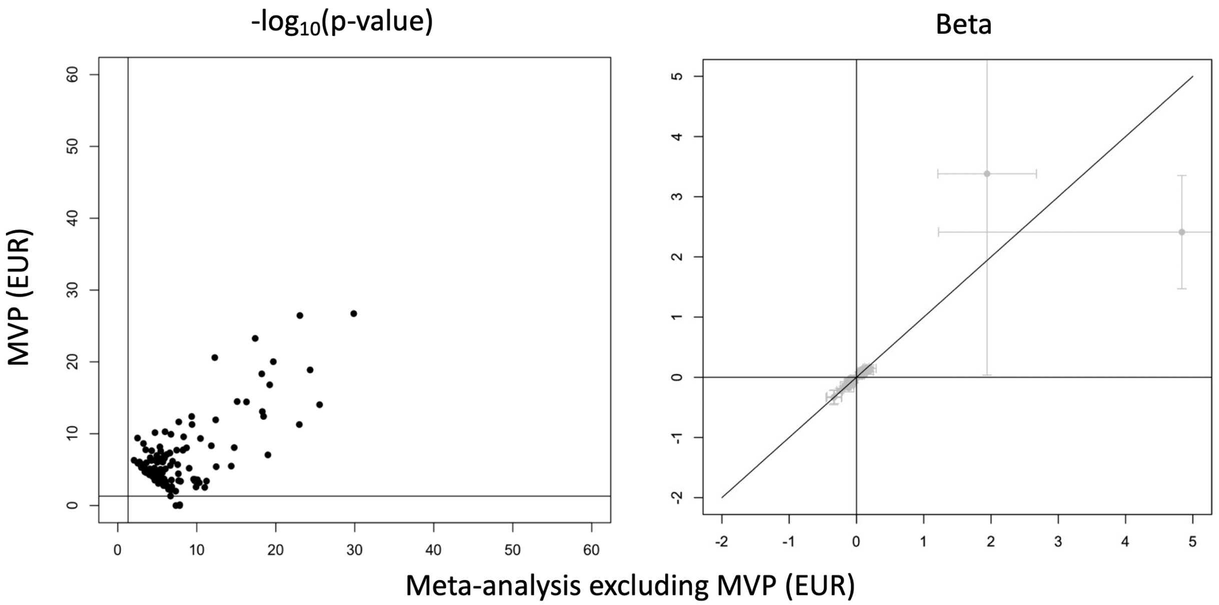

Supplementary Figure 5: Comparison of GWAS p-values (two-sided) in 126 genome-wide significant index variants between MVP (EUR) (Y-axis; N_{case}=17,672; $N_{control}$=303,695) and meta-analysis excluding MVP (EUR) (X-axis; N_{case}=21,549; $N_{control}$=782,412). Three index variants have p-value > 0.05 in MVP (EUR) study. Comparison of effect estimate/beta (95% CI) in the rest of the 123 index variants.

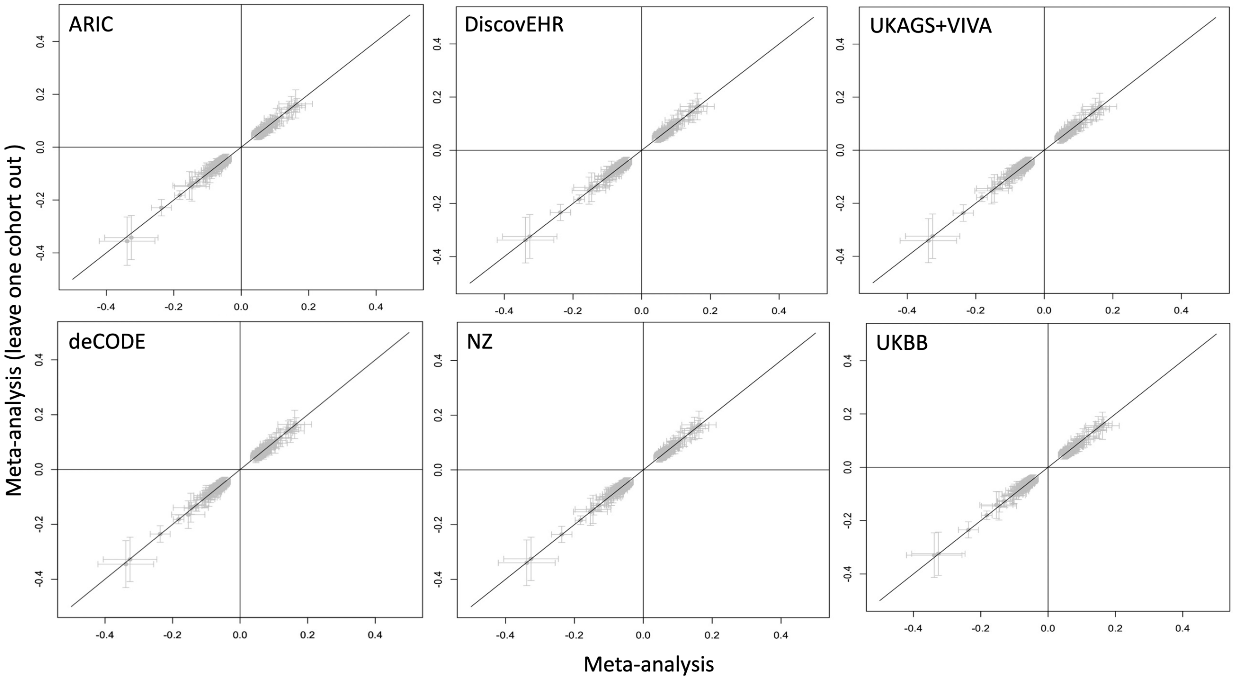

Supplementary Figure 6: Comparison of effect estimates (95% CI) for 121 index variants between AAAgen GWAS meta-analysis (X-axis; N_{case}=39,221; $N_{control}$=1,086,107) and leave-one-cohort-out meta-analysis for six discovery cohorts (Y-axis). The sample sizes in leave one cohort out meta-analyses can be obtained by subtracting numbers in discovery cohorts in **Supplementary Table 1** from AAAgen GWAS meta-analysis.

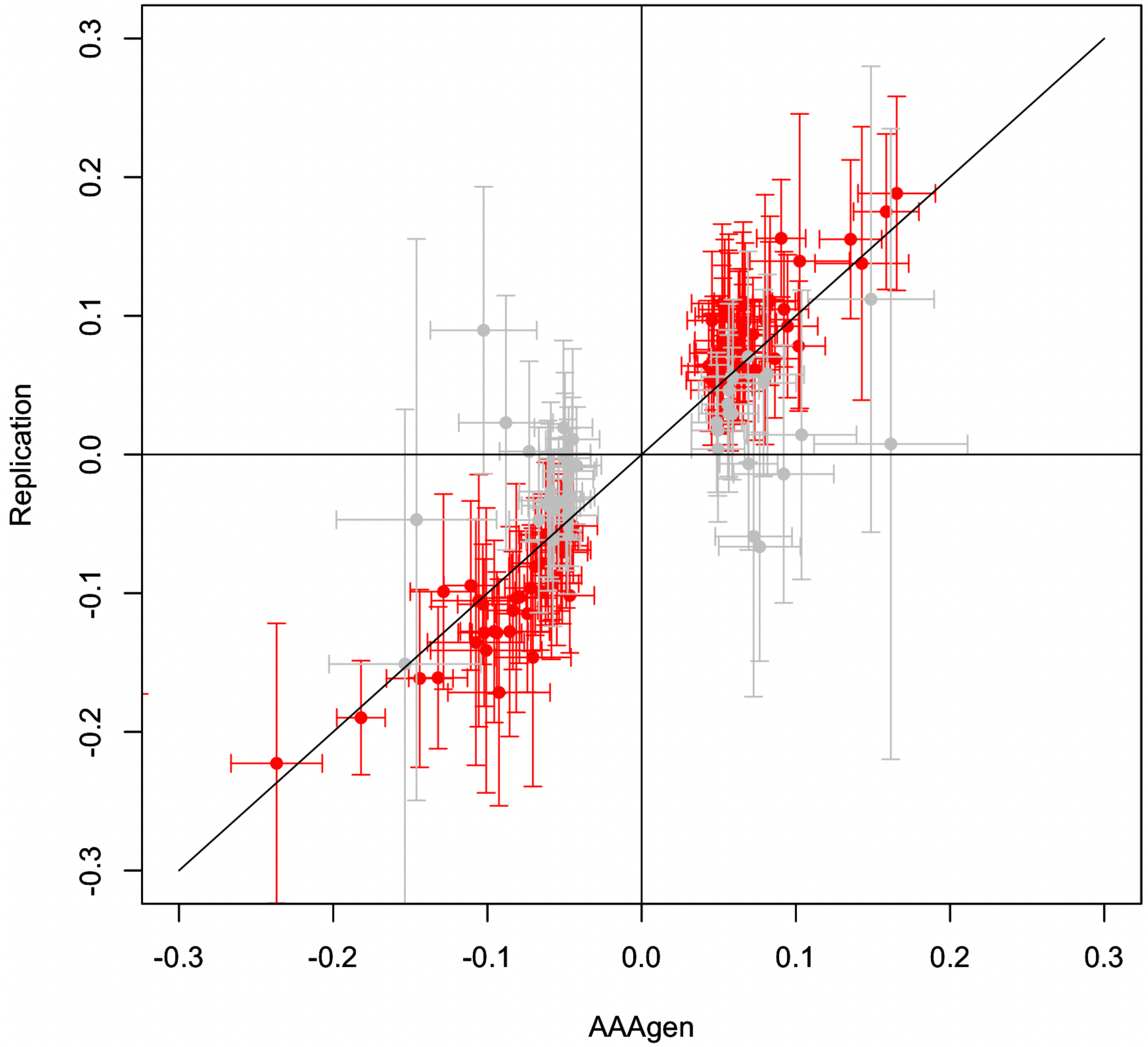

Supplementary Figure 7: Comparison of effect estimates (95% CI) in 121 loci between AAAgen GWAS meta-analysis (X-axis; N_{case}=39,221; $N_{control}$=1,086,107) and external replication cohorts (Y-axis; Max N_{case}=5,451; $N_{control}$=299,885; not all variants were present in 3 external replication cohorts; see **Supplementary Table 2** for further details). The red dots represent 80 variants with two-sided p-value<0.05 in external cohorts and the grey dots represent other 41 variants.

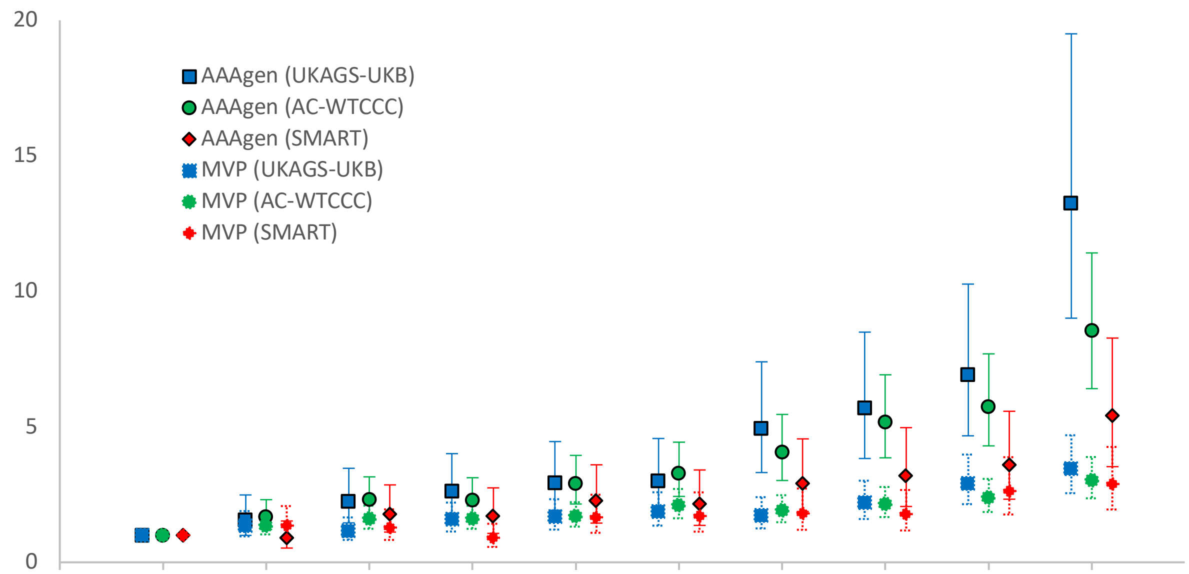

		Score deciles:	1*	2	3	4	5	6	7	8	9	10
AAAgen	UKAGS-UKB	n AAA	32	49	68	78	86	88	134	150	174	271
		n control	662	645	626	616	608	606	560	544	520	423
		OR (95% CI)	Ref	1.6 (1.0-2.5)	2.2 (1.5-3.5)	2.6 (1.7-4.0)	2.9 (1.9-4.5)	3.0 (2-4.6)	5.0 (3.3-7.4)	5.7 (3.8-8.5)	6.9 (4.7-10.3)	13.3 (9-19.5)
	AC-WTCCC	n AAA	68	107	140	139	168	184	215	253	271	342
		n control	665	625	592	594	564	548	518	479	461	391
		OR (95% CI)	Ref	1.7 (1.2-2.3)	2.3 (1.7-3.2)	2.3 (1.7-3.1)	2.9 (2.2-3.9)	3.3 (2.4-4.4)	4.1 (3-5.5)	5.2 (3.9-6.9)	5.7 (4.3-7.7)	8.6 (6.4-11.4)
	SMART	n AAA	31	28	52	50	64	61	78	84	92	123
		n control	346	349	325	326	313	316	298	293	285	254
		OR (95% CI)	Ref	0.9 (0.5-1.5)	1.8 (1.1-2.9)	1.7 (1.1-2.7)	2.3 (1.4-3.6)	2.2 (1.4-3.4)	2.9 (1.9-4.6)	3.2 (2.1-5)	3.6 (2.3-5.6)	5.4 (3.5-8.3)
Klarin et al.	UKAGS-UKB	n AAA	66	86	76	99	104	114	107	130	163	185
		n control	628	608	618	595	590	580	587	564	531	509
		OR (95% CI)	Ref	1.3 (1-1.9)	1.2 (0.8-1.7)	1.6 (1.1-2.2)	1.7 (1.2-2.3)	1.9 (1.4-2.6)	1.7 (1.3-2.4)	2.2 (1.6-3)	2.9 (2.1-4)	3.5 (2.6-4.7)
	AC-WTCCC	n AAA	116	148	170	170	178	207	194	211	227	266
		n control	617	584	562	563	554	525	539	521	505	467
		OR (95% CI)	Ref	1.3 (1-1.8)	1.6 (1.2-2.1)	1.6 (1.2-2.1)	1.7 (1.3-2.2)	2.1 (1.6-2.7)	1.9 (1.5-2.5)	2.2 (1.7-2.8)	2.4 (1.9-3.1)	3 (2.4-3.9)
	SMART	n AAA	43	56	53	39	66	68	71	70	95	102
		n control	334	321	324	337	311	309	305	307	282	275
		OR (95% CI)	Ref	1.8 (1.4-0.9)	2.1 (1.3-0.8)	2.1 (0.9-0.6)	2.2 (1.6-1.1)	2.7 (1.7-1.1)	2.5 (1.8-1.2)	2.8 (1.8-1.2)	3.1 (2.6-1.8)	3.9 (2.9-1.9)

Supplementary Figure 8: Performance of the PRS constructed by this AAAgen GWAS meta-analysis was compared with the Klarin et al. PRS (MVP)[2], the largest GWAS of AAA previously published. Three validation datasets were used. The UKAGS-UKB dataset is a comparison of screen-detected AAA from the UK AAA screening programmes with controls from UK Biobank. The AC-WTCCC data is a case-control study of AAA. The SMART study is an observational cohort of patients presenting with cardiovascular disease and represents a case-control study where controls had prevalent cardiovascular disease. Three different shapes were used to denote odds ratio (95% CI) observed three validation datasets. We observed higher odds ratios by AAAgen compared to Klarin et al. in all validation datasets. *Decile 1 is the reference decile against which all other deciles were compared.

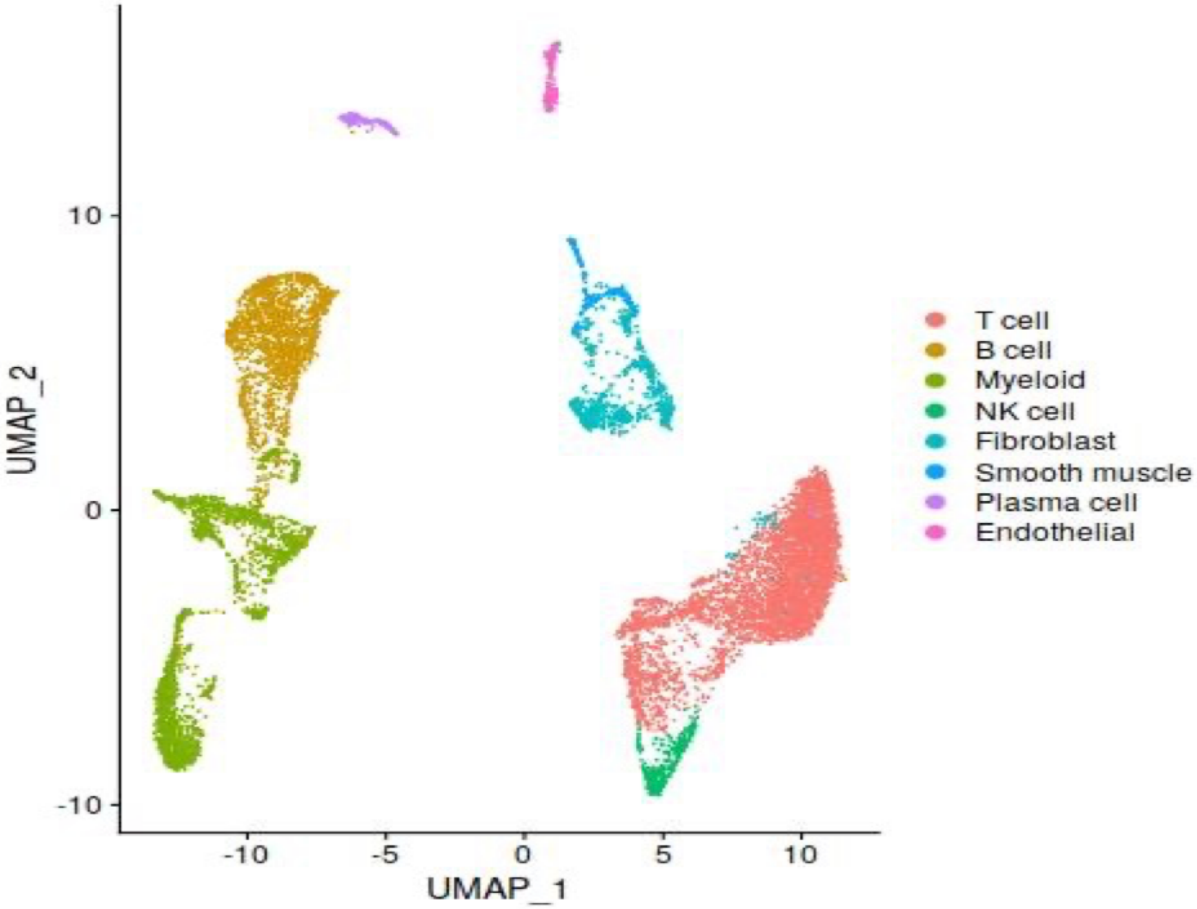

Supplementary Figure 9: UMAP visualization of single cell RNA-seq of aorta used for cell-type enrichment analysis.

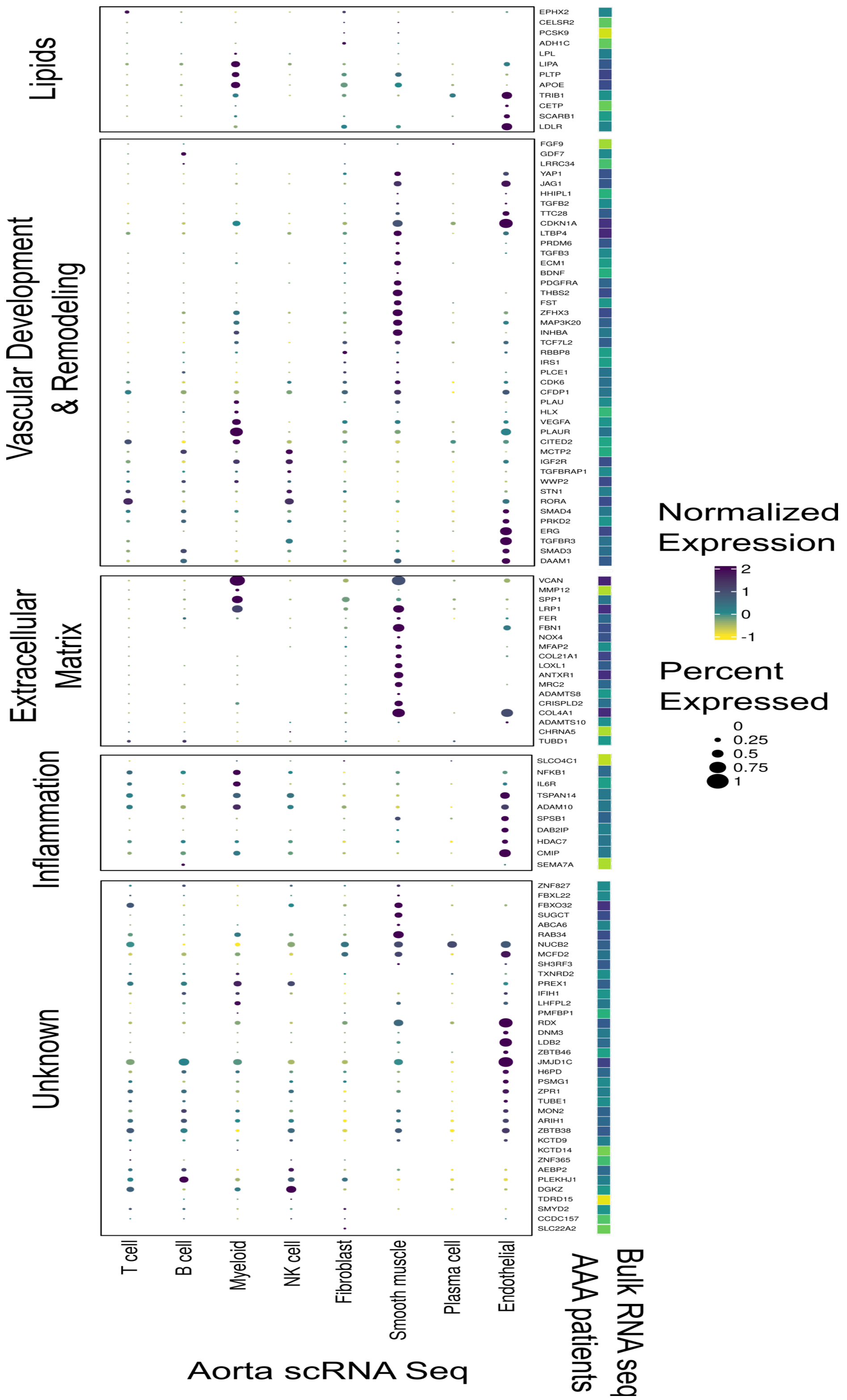

Supplementary Figure 10: Expression of prioritized genes in bulk RNA-seq of 15 AAA patients (right column) and in scRNA seq of aorta (dots in the matrix).

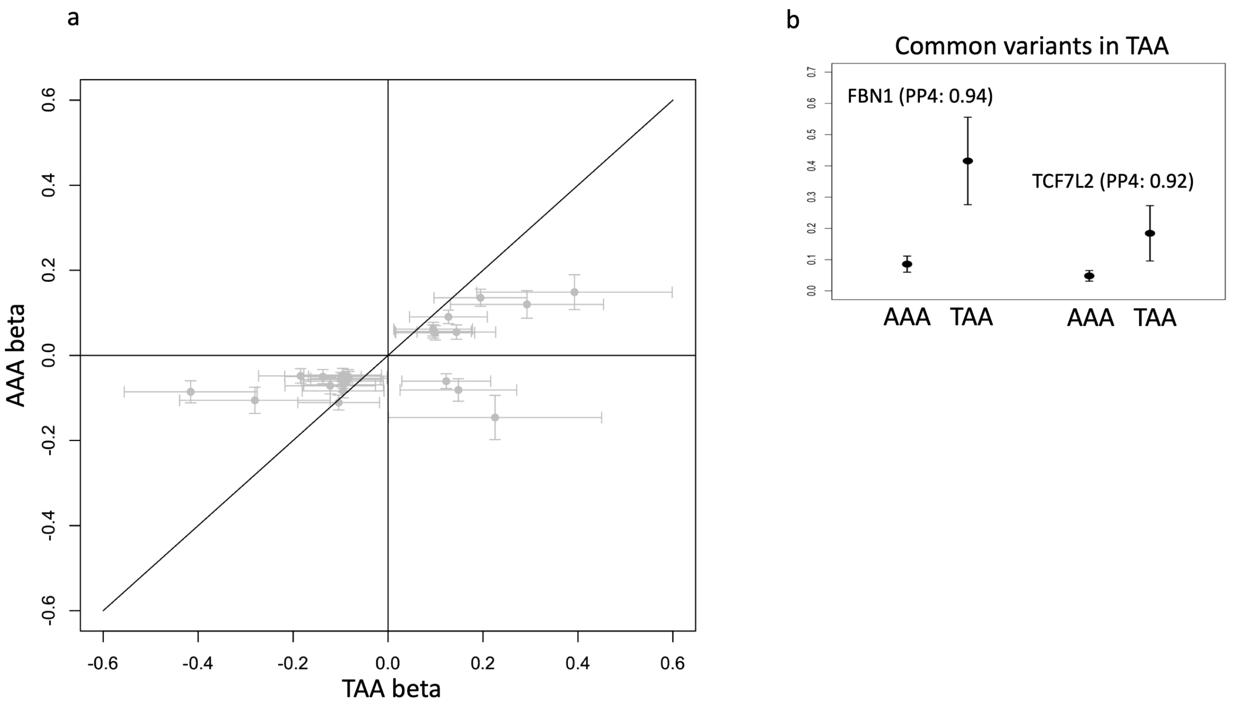

Supplementary Figure 11: a) Comparison of effect estimates /beta (95% CI) in 24 AAAgen GWAS meta-analysis (Y-axis; N_{case}=39,221; $N_{control}$=1,086,107) index variants that were observed to have p-value < 0.05 in TAA GWAS[3] (X-axis; N_{case}=1,351; $N_{control}$=18,295). **b)** Comparison of effect estimates (95% CI) in 2 AAAgen GWAS meta-analysis index variants that were observed to have p-value < 5×10^{-8} in TAA GWAS. All p-values are two-sided. PP4 is the posterior probability of the same causal variant in two traits by colocalization.

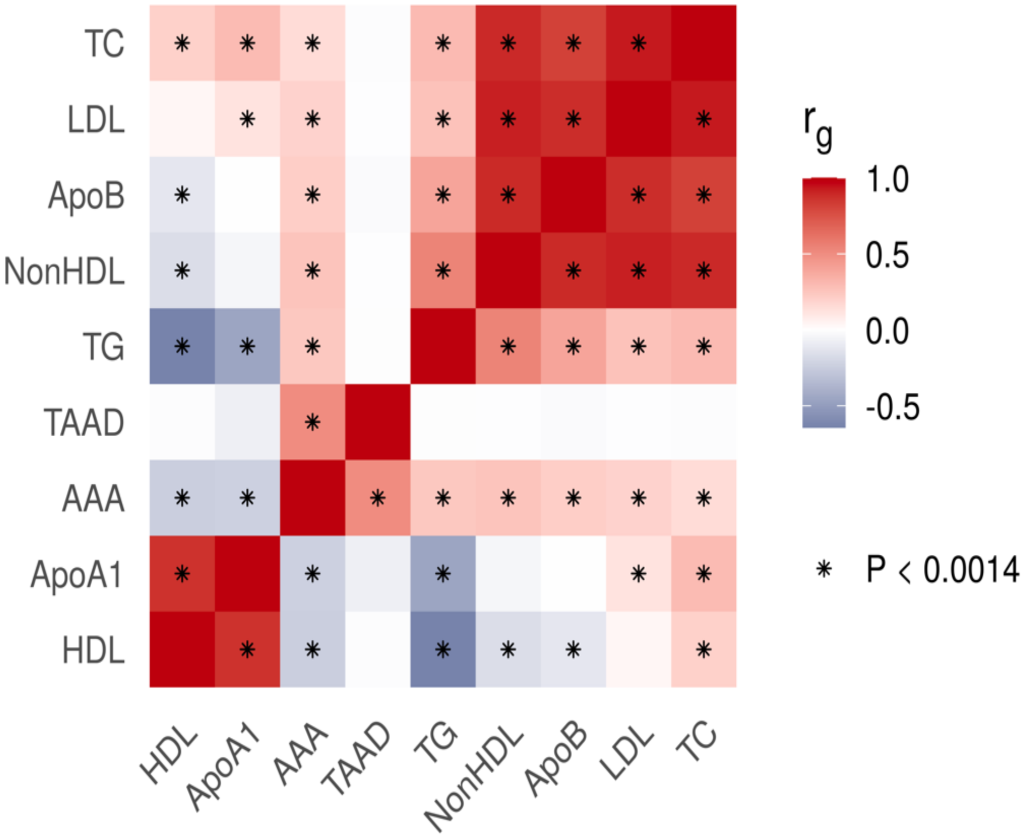

Supplementary Figure 12: Genetic correlation (r_g) between thoracic/abdominal aortic aneurysm and lipid traits. Two-sided p-values were adjusted for 36 pairwise comparisons (*indicates significant genetic correlation with p-value< 0.05/36=0.0014).

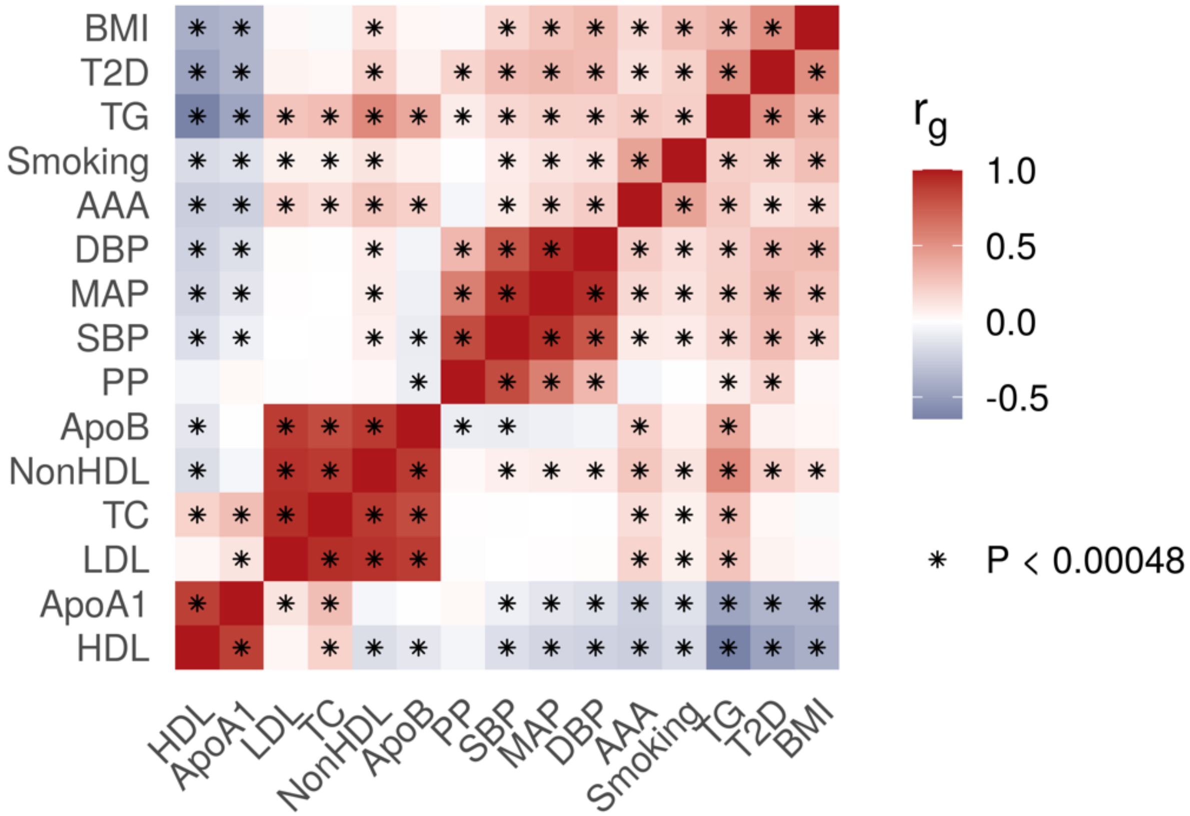

Supplementary Figure 13: Genetic correlation (r_g) between AAA and cardiometabolic traits. Two-sided p-values were adjusted for 105 pairwise comparisons. (*indicates significant genetic correlation with p-value< 0.05/105=0.00048)

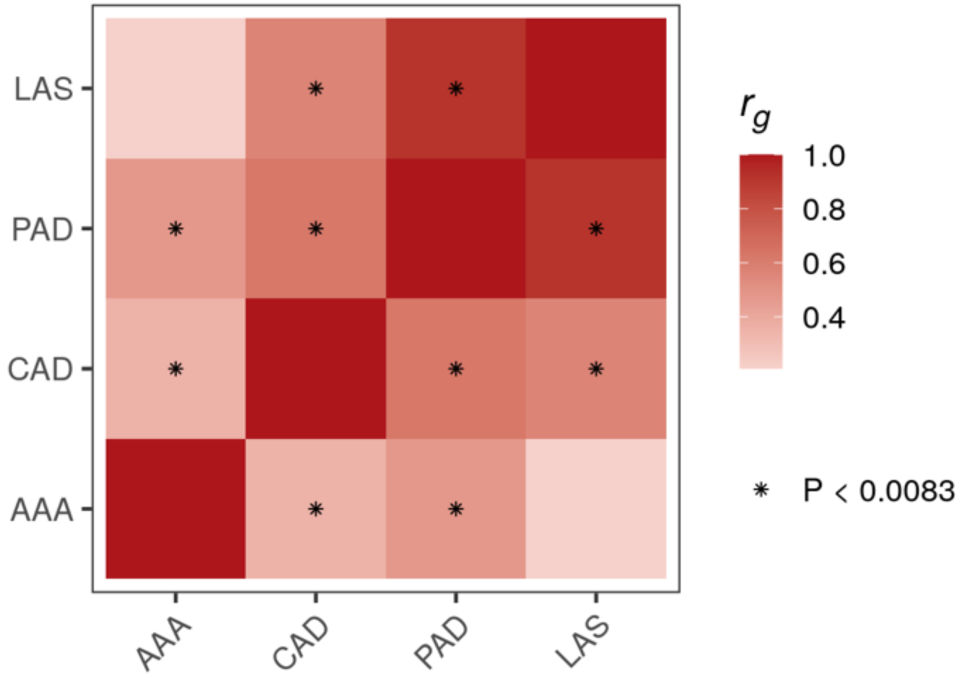

Supplementary Figure 14: Genetic correlation (r_g) between AAA and cardiovascular diseases (CAD: coronary artery disease; PAD: peripheral artery disease; LAS: large artery stroke). Two-sided p-values were adjusted for 6 pairwise comparisons. (*indicates significant genetic correlation with p-value< 0.05/6=0.0083)

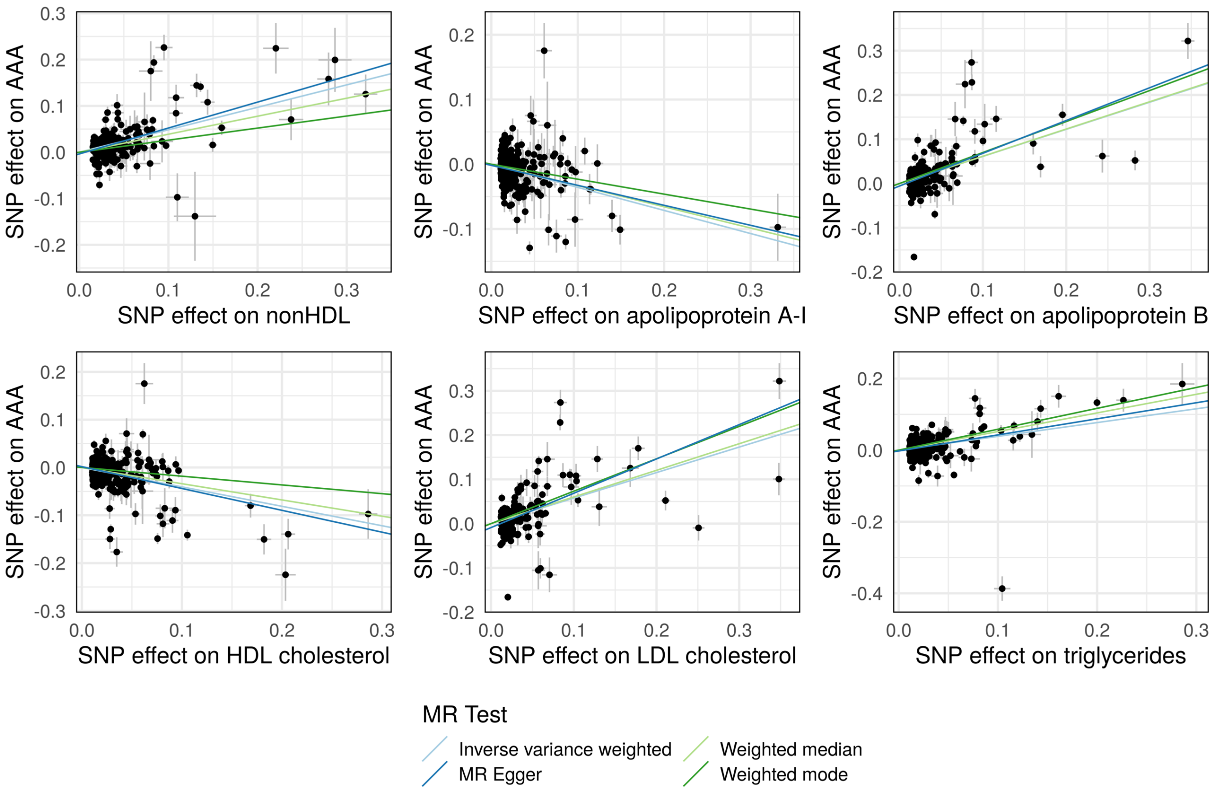

Supplementary Figure 15: Genetic instruments for each lipoprotein-related trait were obtained from GWAS performed with UK Biobank participants including up to 356,284 individuals. Each point represents the effect of each SNP on AAA (y-axis) and each lipoprotein trait (x-axis). Bars represent standard errors. The slope of each colored line represents the estimated effect of each lipoprotein trait on AAA using MR models which make varying assumptions about the presence of pleiotropy and invalid genetic instruments.

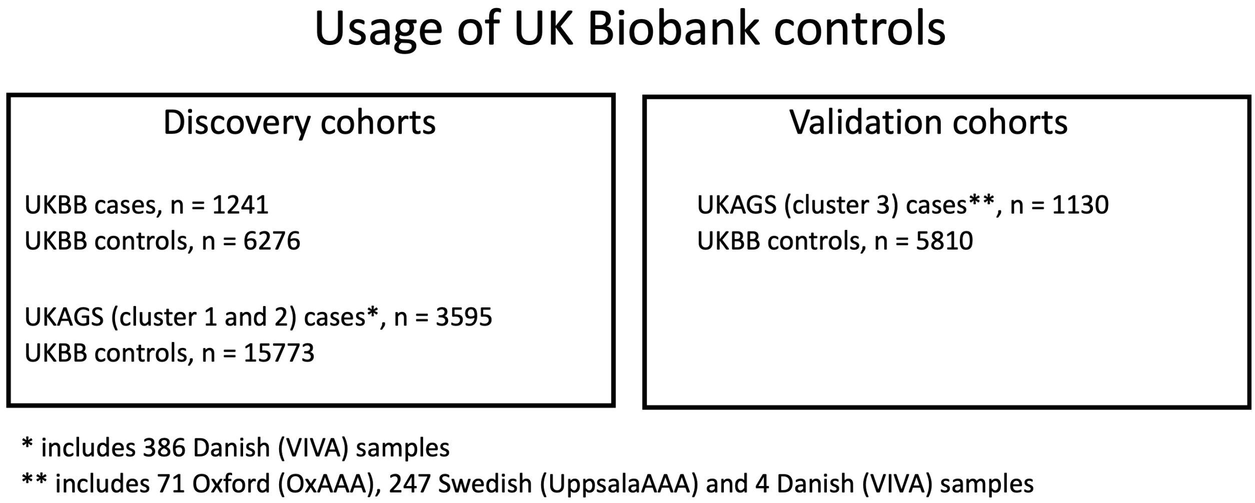

Supplementary Figure 16: Diagram for the usage of UK Biobank controls.

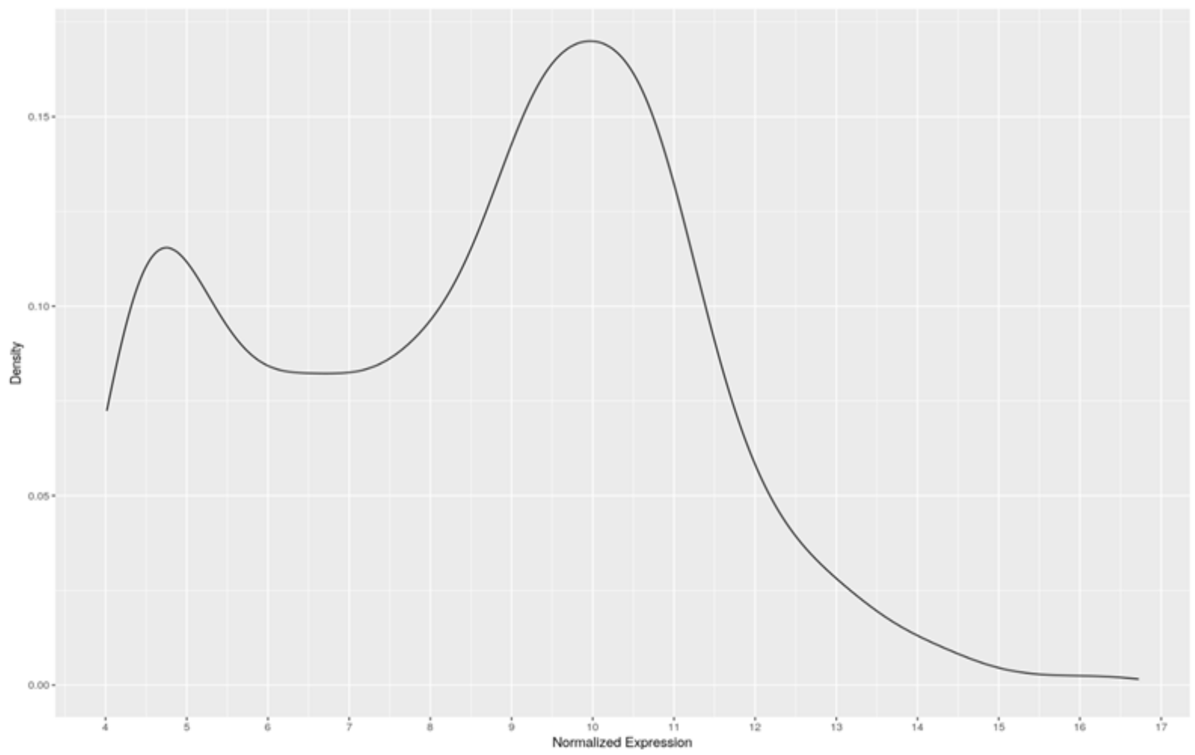

Supplementary Figure 17: Presence or absence of aortic gene expression from bulk RNA-seq of 15 AAA patients was decided based on this bimodal distribution of VST normalized expression (see methods).

Supplementary Note

Supplementary Results

Gene-prioritization: We used 8 indicators to prioritize a single putative causal gene at the 121 genome-wide significant loci. Evidence of these indicators were applied in two stages so that each prioritized causal gene was supported by at least one indicator from stage 1.

In stage 1, candidate genes were selected using the following indicators: 1) protein altering variant (**Supplementary Table 2**); 2) evidence of eQTL using colocalization or transcriptome-wide association study (TWAS) (**Supplementary Table 6-7**); 3) Polygenic Priority Score (PoPS)[4], a similarity-based method that leverages publicly available functional genomics resources. **(Supplementary Table 8)**; 4) evidence of causing a relevant monogenic phenotype using ClinVar database[5] and Renard et al.[6] (**Supplementary Table 9-10**); 5) the gene that is closest to the index variant (**Supplementary Table 2**). In total, we obtained 523 candidate genes from the 121 loci using 5 above indicators in stage 1. In stage 2, these 523 genes were queried in 3 other datasets to obtain evidence of additional indicators, including: 1) differential gene-expression in mouse model of AAA (**Supplementary Table 11**); 2) differential gene-expression in human AAA (**Supplementary Table 12**); 3) relevant phenotypes observed in mouse knock-outs for candidate genes (**Supplementary Table 13**).

With these 8 indicators from stage 1 and 2, the following rules were used in order of precedence to prioritize single likely causal genes in 121 loci (**Supplementary Table 14**). 1) At 21 loci, genes with protein-altering variants were prioritized. 2) At 52 loci, we could assign a single likely causal gene by consensus i.e., a single gene was supported by more indicators (minimum 3) than other genes at that locus. At 11 loci, we observed more than 1 gene with same number of indicators ($\geq$3); in this instance we used distance from index variant as a tie-breaker to prioritize single genes at these loci (e.g. *SMAD3* was prioritized over *SMAD6*). The above-mentioned steps prioritized a single gene at 84 loci. **Figure 3b** summarizes the support of various indicators for these 84 genes. 3) In the remaining 37 loci with no clear consensus candidate gene, we prioritized the closest gene from the index variant as the likely causal gene, as this has been shown to perform reasonably well (~65-70%) in recent studies[7,8].

Based on GO molecular function annotations, we observed enrichment of genes related to lipid metabolism, including genes involved in binding lipoprotein particle/receptor(s) (*APOE, LDLR, LPL, PCSK9, PLTP, SCARB1*). We also observed enrichment of TGF-β related genes (*TGFBR3, TGFB2, SMAD3, TGFB3, TGFBRAP1, LTBP4, GDF7*) that are likely involved in vascular development. TGF-β related genes, as well as several other prioritized genes (*IL6R, VEGFA, INHBA, IL1F10*), are also involved in cytokine activity and receptor binding, as key modulators of inflammatory mechanisms. We observed 7 genes that encode transcription factors (*HDAC7, LDB2, RBBP8, SMAD3, TCF7L2, WWP2, YAP1*) and 3 additional transcriptional co-activators (*RORA, SMAD4, TERT*). A study of murine and human cardiac fibrosis determined that *WWP2* was a master regulator expressed in diseased human heart to turn on pro-fibrotic and extracellular matrix genes and was induced by TGF-β and SMAD related pathways[9]. Other studies showed that *YAP1* is a central regulator of phenotypic switching of vascular smooth muscle cells (VSMCs)[10] and negatively regulates differentiation of VSMCs from cardiovascular progenitor cells by decreasing transcription of myocardin[11]. Eight prioritized genes were highlighted for growth factor activity/receptor binding (*JAG1, VEGFA, FGF9, BDNF, INHBA, PDGFRA, FER, IL6R)* which are known to be involved in vascular remodeling. The most significantly enriched GO cellular component term was the collagen-containing extracellular matrix (*TGFB2, ECM1, TGFB3, LTBP4, THBS2, LOXL1, ADAMTS10, F7, VCAN, COL4A1, MFAP2, COL21A1, APOE, ADAMTS8, FBN1)* indicating significant involvement of extracellular matrix dysregulation in AAA pathogenesis.

Supplementary Methods

Discovery cohorts:

ARIC: The Atherosclerosis Risk in Communities (ARIC) Study is a population-based cohort recruited from four US communities in North Carolina, Mississippi, Minnesota and Maryland. ARIC identified incident, clinical AAAs by searching hospitalization and death records as well as Medicare data through 2011. All participating institutions gave institutional review board approval for this study and all participants provided written informed consent. Clinical AAA were defined as those who had a hospital discharge diagnosis from any of the above sources, or

two Medicare outpatient claims that occurred at least one week apart, with *ICD-9-CM* codes of 441.3 or 441.4, or procedure codes of 38.44 or 39.71, or the following cause of death codes: *ICD-9* 441.3 or 441.4 or *ICD-10* code I71.3 or I71.4[12,13]. AAAs based on procedure codes were required to be verified by diagnosis codes. Thoracic, thoracoabdominal, or unspecified aortic aneurysms were treated as non-events. Participants reporting prior AAA surgery or aortic angioplasty at baseline were excluded. Genotyping was performed with Affymetrix Genome-Wide Human SNP array 6.0 and race-specific imputation of variant dosages to the 1000 Genomes Project Phase I version 3 reference panel was performed with IMPUTE2. Association analysis was performed using logistic regression in SNPTEST[14] among 408 AAA cases and 8554 non-AAAs of EUR ancestry. Covariate adjustment included age at baseline, sex, pack-years of smoking, and principal components (PC) 1-5.

CHB-CVDC and DBDS: Copenhagen Hospital Biobank (CHB) is a hospital driven biobank and includes leftover EDTA blood samples drawn for blood type testing or red cell antibody screening from hospitalized patients in the Danish Capital Region[15]. In addition to genetics, EHR, national socioeconomic and health registries extensively characterize each patient. Patients with AAA are included under the Copenhagen Hospital Biobank Cardiovascular Study (CHB-CVDC)[16]. The healthy blood donors from "The Danish Blood Donor Study" (DBDS) are included in the study as controls[17]. Both CHB-CVDC and DBDS have been approved by the National Committee on Health Research Ethics (NVK-1708829 and NVK-1700407) and the Danish Capital Region Data Protection Office (P-2019-93 and P-2019-99). AAA cases were identified using the following ICD codes (ICD8: 44120/44121/44129; ICD10: I71.3/I71.4). Individuals with aorta dissection were not included in the analysis. AAA cases were compared to the remaining individuals from the DBDS and CHB-CVDC studies excluding individuals with abdominal aortic aneurysm, thoracic aortic aneurysm and intracranial aneurysm using the following ICD codes (ICD8: 44109/44111/44110/44119/44120/44121/44129/44299/43000/43001/43008/43009/43090/43091/43098/43099/43701/43791; ICD10: I71.0/I71.1/I71.2/I71.3/I71.4/I71.5/I71.6/I71.8/I71.9/I72/I60/I67.1). The Infinium Global Screening Array from Illumina was used for genotyping samples from CHB-CVDC and DBDS. Whole-genome sequence data from 8429 Danes along with 7146 samples from North-Western

Europe forms a reference panel backbone used for imputation[18]. The association analysis of 3,079 AAA cases and 180,236 controls was performed with SAIGE[19] using year of birth, sex and 10 PCs as covariates.

CHIP+MGI: The Cardiovascular Health Improvement Project (CHIP) is a cohort of individuals treated at Michigan Medicine with linked genotype, EHR, and family history data. The Michigan Genomics Initiative (MGI) is a hospital-based cohort with linked genotype and EHR data from participants recruited during pre-surgical encounters at Michigan Medicine. Both studies were approved by the Institutional Review Board of the University of Michigan Medical School (IRBMED) (HUM00052866, HUM00071298) and informed consent was obtained from study participants. 534 cases from CHIP were identified as aneurysm in abdominal aorta following diagnosis by Cardiologists and after excluding cases with known dissection. From MGI, 749 cases were identified using ICD codes (ICD9: 441.3/4441.4; ICD10: I71.3/I71.4) after excluding cases with known dissection (ICD9: 441.00-03; ICD10: I71.00-03). After removing samples with related phenotypes (phecodes 440–449.99), a case-control matching strategy was used to identify controls from MGI. Case-control matching was performed using MatchIt package[20] in R using birth year, gender, array version and 4 genotype PCs. Samples in CHIP and MGI were genotyped using two versions of customized Illumina Infinium CoreExome-24 bead arrays: UM_HUNT_Biobank_11788091_A1 and UM_HUNT_Biobank_v1-1_20006200_A and imputation. Imputation was performed with the Haplotype Reference Consortium (HRC) panel using Minimac4[21]. Association analysis of 1,283 AAA cases and 12,202 controls was performed using SAIGE[19] with birth year, gender, array version and 4 genotype PCs as covariates.

deCODE: Icelandic individuals with AAA were identified from a registry of individuals, using ICD codes (ICD9: 441.3, 441.4, ICD10: I71.3, I71.4) who were admitted at Landspitali University Hospital, in Reykjavik, Iceland, 1980–2016, or diagnosed at private clinic in Reykjavik, Iceland. The Icelandic controls used were selected from individuals who have participated in various GWA studies and who were recruited as part of genetic programs at deCODE. Individuals with known cardiovascular disease were excluded as controls but controls were unscreened for AAA. Genotyping was performed using various Illumina SNP chips and phased using long-range phasing[22], followed by imputation of 37.6m high-quality variants from 49,962 whole genome-sequenced Icelanders[23,24]. The association analysis of 1,656 AAA cases and 265,410 controls was

performed using deCODE genetics software[23], and we adjusted for gender, county of origin, current age or age at death (first and second order term included), blood sample availability for the individual, and an indicator function for the overlap of the lifetime of the individual with the time span of phenotype collection. We used LD score regression to account for distribution inflation due to cryptic relatedness and population stratification[25].

DiscovEHR: The DiscovEHR cohort is a collaborative effort between Geisinger Health System and the Regeneron Genetics Center. DiscovEHR participants are from Geisinger Health System's MyCode Community Health Initiative including patients from rural Pennsylvania (USA) recruited from 2007-2021. The GHS MyCode initiative and the DiscovEHR study were approved by the Geisinger Institutional Review Board (2006-0258). We included individuals with EHR data, genotype data, and those determined to be of primarily European descent based on SNP-derived principal components analysis. We defined AAA cases based on the presence of at least two instances of any of the following ICD10 codes: 441.3, 441.4, I71.3, I71.4, and required controls to lack any occurrence of the aforementioned ICD10 codes, in addition to the ICD10 codes I71-75, I77-79, K55. We further removed individuals who contributed to the study via the eMERGE consortium. DiscovEHR participants were genotyped on either the Illumina Omni Express Exome or Global Screening Array and imputed from the HRC reference panel using the Michigan Imputation Server. We performed association analysis of 2,238 AAA cases and 105,433 controls using whole genome regression in REGENIE (v1.0), including Age, Age^2, Sex, Age×Sex, Age^2×Sex, 10 common variant (MAF>1%) derived principal components, and an array batch indicator as covariates in the model.

eMERGE: The electronic Medical Records and Genomics (eMERGE, Phase 3) network is a consortium with EHR and genetic data on ~100,000 patients from 12 institutions across the US, including ~20% samples from African ancestry. Samples with 2 or more instances of ICD9 codes 441.3/4441.4 or ICD10: I71.3/I71.4 were defined as cases and samples without instance of case ICD codes were defined as controls. Case control matching was performed separately on EUR and AFR samples to retain 5 controls per each case matching based on age and sex using MatchIt R program. Samples in eMERGE Phase III were genotyped on 78 different Illumina and Affymetrix SNP array platforms. Data from different sites and genotyping platforms were imputed using the HRC reference panel on Michigan Imputation Server and then merged into one set[26,27].

We identified 3092 cases and 15,025 controls in the EUR dataset and 119 cases and 603 controls in the AFR dataset, and logistic regression was performed on unrelated samples within ancestry groups using PLINK 2.0. All models were adjusted by clinical site and first 5 principal components.

HUNT: The Trøndelag Health (HUNT) Study[28] is a population-based health study from the Trøndelag region in Norway. Individuals have been enrolled in the study though multiple phases (HUNT1, HUNT2, HUNT3, and HUNT4) beginning in 1984. During each enrollment period, individuals living in the region who are 20 years or older are invited to participate. To date, over 120,000 individuals have participated and have longitudinal health record data available from surveys, medical records, and biological samples. AAA cases were defined by ICD-9 codes 441.3 and 441.4 and ICD-10 codes I71.3 and I71.4. HUNT samples were genotyped using the Illumina HumanCoreExome array and imputed with Minimac3[21] using a combined reference panel consisting of the HRC v1.1 combined with whole genome sequencing of 2,201 HUNT participants. Association testing of 734 cases and 68,901 controls was performed using SAIGE[19] with batch, sex, birth year, and PC1-4 as covariates.

Mayo VDB: Mayo Vascular Disease Biorepository (VDB) at the Mayo Clinic was established to archive DNA, plasma, and serum from patients with suspected atherosclerotic cardiovascular disease (ASCVD) referred for noninvasive vascular evaluation and exercise stress testing at the Mayo Clinic Gonda Vascular Center from January 14, 2006 to July 24, 2020. ASCVD phenotypes and related comorbidities were ascertained using previously validated electronic phenotyping algorithms based on both structured data elements (ICD diagnosis codes, CPT codes, and laboratory measurements) and natural language processing of unstructured data elements, such as vascular laboratory and imaging reports in the EHR. The study was approved by the Mayo Clinic Institutional 74 Review Boards (IRB # 08–008355). After exclusion of overlapping samples between Mayo VDB Dataset and eMERGE III V3 Imputed Array Dataset[26], AAA cases were defined as having an infrarenal abdominal aortic diameter ≥3 cm or a history of open or endovascular AAA repair based on an electronic phenotyping algorithm using Natural Language Processing on ultrasound reports, validated by manual chart review. Controls were not known to have AAA and had no ICD-9 diagnosis codes for AAA. Genotyping was performed using three different Illumina platforms, including Illumina Human660W-Quad V1,

HumanCoreExome Beadchip, and Human 610 Quad V1 platforms. We combined samples genotyped on different platforms by genotype imputation based on a HRC v1.1 panel. Association testing of 771 AAA cases and 4,913 controls was performed on the imputed dataset using SAIGE software with the following covariates: study enrollment age, sex, genotyping platform, first 5 principal components of ancestry.

MVP: In the Million Veteran Program (MVP), individuals aged 18 to over 100 years have been recruited from 63 VA Medical Centers across the United States. MVP received ethical/study protocol approval by the VA Central Institutional Review Board, and informed consent was obtained for all participants. Each additional study received approval from their local institutional review board. From the participants passing quality control in MVP, individuals were defined as having AAA or being a disease-free control using a previously utilized[2] definition initially proposed by Denny et al[29]. AAA cases were defined as the presence of two instances of any of the following ICD-9/10 codes in a participant's EHR: 441.3, 441.4, I71.3, I71.4. Controls were defined as possessing zero occurrences of the aforementioned ICD codes, as well as zero occurrences of the ICD-9 codes 440-448, or ICD-10 codes I71-75, I77-79, K55. In our MVP analysis, we evaluated 17,672 AAA cases and 303,695 controls of European ancestry, and 1,888 AAA cases and 87,728 controls of African ancestry. Genotyped and imputed DNA sequence variants in individuals of were tested for association with AAA using logistic regression adjusting for age, sex, and 5 principal components of ancestry assuming an additive model using the PLINK2.0 statistical software program.

NZ AAA Genetics Study: The Vascular Research Consortium of New Zealand recruited New Zealand men and women with a proven history of AAA (infra-renal aortic diameter ≥ 30 mm proven on ultrasound or CT scan). Approximately 80% had undergone surgical AAA repair (typically AAA's > 50-55 mm in diameter). The vast majority of cases (>97%) were of Anglo-European ancestry. The control group underwent an abdominal ultrasound scan to exclude (>25 mm) concurrent AAA and Anglo-European ancestry was required for inclusion. Controls were also screened for peripheral artery disease (PAD; using ankle brachial index), carotid artery disease (ultrasound) and other cardiovascular risk factors. All participants were genotyped in two separate case-control cohorts using the Affymetrix SNP6 (cohort 1; 608 AAA cases and 612 controls) or Illumina Omni2.5 (cohort 2; 397 cases and 384 controls) GeneChip arrays and had call rates >95%

(mean 99.2%). Imputation was then conducted using IMPUTE 2.2 run on the BCISNPmax database platform (version 3.5, BCI Platforms, Espoo, Finland). The reference haplotypes were based on the 1000 Genomes June 2011 release. Imputed calls were filtered by quality score (>0.9) to restrict to higher quality imputed SNPs. The genomic inflation factors were 1.06 and 1.05 respectively (MAF >0.05, 5.4 million SNPs). Case-control genome wide association analyses were conducted using PLINK (version 1.07).

PMBB: Penn Medicine biobank (PMBB) recruits patients from throughout the University of Pennsylvania Health System for genomic and precision medicine research. Participants actively consent to allow the linkage of biospecimens to their longitudinal EHR. Currently, >60 000 participants are enrolled in the PMBB. A subset of ~45000 individuals who have undergone whole exome sequencing and genotyping, performed through a collaboration with the Regeneron Genetics Center. A further subset of ~23000 subjects with imputed genotype data was used in this analysis. 19,515 recruits were genotyped on three different arrays, Illumina Quad Omni, Global Screening Assay v1 and Global Screening Assay v2. Genotype imputations were performed using the Eagle2[30] and Minimac softwares[21] on the Michigan Imputation server and were completed for all autosomes with the HRC reference panel. AAA cases were found using the AAA phecode definition of 442.11, meaning at least 1 encounter with abdominal aortic aneurysm diagnosis codes and excluding other confirmed disease of the arteries. Controls were defined as all others with genotype data and no evidence of AAA. GWAS of 388 cases and 9879 controls was performed using plink software with age at recruitment, gender, genetic determined ancestry and the first 10 principal components.

TABS: The Triple A Barcelona Study (TABS) is a hospital-based study recruiting individuals with AAA treated in the Hospital de la Santa Creu i Sant Pau in Barcelona, Spain. All individuals have repetitive measurements of the abdominal aortic diameter, either by CT-scan or by ultrasound along with anthropometric and clinical information. DNA, RNA, and plasma samples were collected from all individuals. All participants gave written informed consent. All procedures were approved by the Institutional Review Board at Hospital de la Santa Creu i Sant Pau. Cases were defined as those with dilation of the abdominal aorta with a diameter higher than 30 millimeters. Type B dissections that progress to AAA, saccular aneurysms or thoracic

aneurysms were excluded. In addition to 42 cases from TABS, 10 cases and 82 controls were leveraged from the Triple A Genetic Study (TAGA)[31]. Also, 401 controls were leveraged from the RETROVE study[32]. Genome-wide genotyping was performed using the Infinium Global Screening Array-24 v2.0 from Illumina (coverage 665,608 variants) and imputed to the HRC reference panel using the Michigan server. Association analysis of 52 AAA cases and 483 controls was performed using SAIGE[19] with birth year, sex, batch and PCs as covariates.

UKAGS+VIVA and UKBB: The UK Aneurysm Growth Study (UKAGS), The Viborg Vascular (VIVA) and UK Biobank (UKBB) cohorts are described together since cases from all three cohorts were compared with controls from UKBB. The UK Aneurysm Growth Study (UKAGS) is a prospective study of men attending the NHS aneurysm screening programmes in the UK at reaching age 65. Men recruited into the UKAGS completed a postal questionnaire to obtain information on smoking, comorbidities and medications. Screening outcomes (ultrasound measured AAA diameter) were obtained directly from the AAA screening programmes. Ethical approval was granted by an NHS research ethics committee. The study was funded by the British Heart Foundation (CS/14/2/30841 and RG/18/10/33842) and the Circulation Foundation. The VIVA screening trial is a randomized, clinically controlled study designed to evaluate the benefits of vascular screening and modern vascular prophylaxis in a population of 50,000 men aged 65-74 years, randomized to either receive an invitation for vascular screening or being a control. Ethical approval was granted by the research ethics committee of Mid Denmark (M20080028), and funded by the FP7, EU, and the Region of Mid Denmark. ClinicalTrials.gov NCT00662480. Individuals included from both studies were of white British/Danish ancestry, with PCA identified outliers excluded; related individuals were identified with PLINK v1.9 IBD computation (--genome), and one of any pairwise kinships removed.

UK biobank (UKBB) is a large prospective study with over 500,000 participants aged 40–69 years when recruited between 2006–2010. UKBB subjects were included for a UKBB-UKBB case-control association study, and to provide control subjects for each of the separate case groups used in the discovery and validation cohorts (see Additional validation cohorts for PRS), matching for sex and age, and without overlap of subjects between analyses. See **Supplementary Figure 16** for the usage of UK biobank controls in various analyses.

To identify AAA cases in UKBB the following ICD and OPCS codes were searched for in the UKBB hospital inpatient data. Any individual with one or more of the following codes was defined as a case: ICD9: 441.3, 441.4; ICD10: I71.3, I71.4; OPCS4: L184, L185, L186, L194, L195, L196, L271, L275, L276, L281, L285, L286. To exclude individuals with any potential aortic pathology, such as thoracic aortic aneurysm, the following ICD codes were used as exclusion criteria for controls: ICD9: 441.00, 441.01, 441.02, 441.03, 441.1, 441.2, 441.5, 441.6, 441.7, 441.9; ICD10: I71.0, I71.1, I71.2, I71.5, I71.6, I71.8, I71.9. The group of controls was then restricted to the UKBB 'in white British ancestry. Batch missing tests (--test-missing in PLINK) were carried out to exclude variants with highly significant difference in missingness between cases and controls (P <0.001).

All participants in UKAGS and VIVA were genotyped using the UKBB Axiom Array. UKBB participants were genotyped using the UKBB Axiom array and UKBB BiLEVE Axiom array. All imputation, including for UKBB was carried out on the Michigan Imputation Server, Minimac 4, 1000G Phase3 v5 (GRCh37/hg19) reference panel (Rsq filter = 0.3)), except for the UKBB-UKBB case-control selection, which made use the available UKBB imputation (UK10K haplotype reference panel + 1000G Phase 3). Association analysis of 3,595 AAA cases (3,209 UKAGS + 386 VIVA) and 15,773 controls (UKBB) was performed with PLINK v2.00a, with following parameters, --geno 0.02, --hwe 1e-8, --maf 0.01. The association test with 1,241 AAA cases (1081 males, 160 females) and 6,276 UKBB controls (5466 males, 810 females) was performed with SNPTEST v2.5.2, with following parameters, -frequentist additive, -method expected.

Additional validation cohorts for PRS: Additional AAA case cohorts from Oxford (UK), Uppsala (Sweden) and Utrecht (Netherlands) became available during the conduct of the study. Those from Oxford and Uppsala were genotyped alongside remaining cases from the UKAGS and VIVA study that had not been included in the discovery study and were analyzed together in comparison to controls taken from UK Biobank. The Aneurysm Consortium AAA GWAS [33] had not met QC criteria for inclusion in the discovery analysis but was available for validation of polygenic risk scores. The UCC-SMART cohort of people with prevalent cardiovascular disease[34] was used for PRS validation to determine discriminatory performance to identify AAA cases amongst populations with cardiovascular disease.

OxAAA: Multimodal Assessment of Aortic Aneurysm Disease Pathogenesis: Oxford Abdominal Aortic Aneurysm Study (OxAAA). Single centre study at the Oxford University Hospitals NHS Trust, funded by the NIHR Oxford Biomedical Research Center. IRAS ID: 122591. REC name: South Central - Oxford C Research Ethics Committee. REC reference: 13/SC/0250.

UppsalaAAA: Uppsala Abdominal Aortic Aneurysm Cohort Study, part of the collection Swedish Cohort Consortium (Cohorts.se). A prospective population-based case-control study which aims to find biomarkers for the cause and progression of AAA and investigate how persons with or without an AAA experience their health. All 65-year-old men, identified through the National Population Registry, were invited to an ultrasound examination. Financial support was provided by the Swedish Research Council (grant 2012-1978), the Swedish Heart and Lung Foundation, the King Gustaf V's and Queen Victoria's Freemason Foundation, and Regional Uppsala-Örebro Research Grant

AC: Aneurysm Consortium (AC), cases of AAA together with available DNA recruited in eight centers in the UK, Australia, and New Zealand and also samples from the United Kingdom Small Aneurysm Trial from inpatient populations, outpatient clinics, or population screening programs in the participating centers. AAA ascertainment (infrarenal aortic diameter of >30 mm) was by either ultrasonography or by cross-sectional imaging except for patients who presented with acute rupture and for whom it was assumed that the AAA was >55 mm. Further details, including funding, ethical approval in Bown et al.[33].

WTCCC2: Wellcome Trust Case Control Consortium 2 data used included samples from the 1958 British Birth Cohort and from the UK National Blood Service. Further details in Bown et al.[33] and https://www.wtccc.org.uk/.

UCC-SMART: The UCC-SMART study is an ongoing, single-centre, prospective cohort at the tertiary referral centre University Medical Center Utrecht (UMCU) in the Netherlands described elsewhere[34].

PRS validation set 1: This set includes 808 cases from UKAGS and 4 cases from VIVA that were not used in the meta-analysis, 247 cases from UppsalaAAA, 71 cases from OxAAA, and

5810 controls from UKBB. All participants in UKAGS, UppsalaAAA, OxAAA and VIVA were genotyped using the UK Biobank Axiom Array. UKBB participants were genotyped using the UK Biobank Axiom array and UK Biobank BiLEVE Axiom array. The following QC filters were used: 1) White British ancestry only, 2) 1 of any pairwise kinships removed, 3) outliers identified by PCA and excluded, 4) batch missing test (--test-missing in PLINK) to exclude variants with highly significant difference in missingness between cases and controls (P <0.001), 5) variants with missing call rates > 2% excluded. A pre-imputation check using the McCarthy Group tool was performed. All imputation, including for UKBB was carried out on the Michigan Imputation Server, Minimac 4, 1000G Phase3 v5 (GRCh37/hg19) reference panel, Rsq filter = 0.3.

PRS validation set 2: This set includes 1887 cases from AC and 5437 controls from WTCCC. The case and control cohorts were separately genotyped with the Illumina 670K BeadChips. Raw intensity data were normalized with BeadStudio, and genotypes were called concurrently from the combined case control data set with the Illuminus algorithm. The following QC filters were used: 1) Identity-by-state (IBS) clustering carried out in PLINK and extreme outlier individuals excluded, 2) Batch missing test (--test-missing in PLINK) used to exclude variants with highly significant difference in missingness between cases and controls (P <0.001), 3) variants with missing call rates > 2% excluded. A pre-imputation check using the McCarthy Group tool was performed. Imputation was carried out on the Michigan Imputation Server, Minimac 4, 1000G Phase3 v5 (GRCh37/hg19) reference panel, Rsq filter = 0.3.

PRS validation set 3: A subset of 6,971 patients from the UCC-SMART study that had been recruited between September 1996 and August 2010 were genotyped using the Illumina GSA array. All SNPs went through a thorough quality control check using PLINK v.1.9. Genotype imputation was performed using IMPUTE2 v2.3.0. After imputation 91.3 million SNPs were available. SNPs with an imputation quality (R2) <0.3, a minor allele frequency below 5%, and SNPs with a Hardy-Weinberg equilibrium p-value <1 x 10-6 were also excluded, resulting in 19.9 million imputed SNPs available for the PRS validation. Patients of non-European ancestry, with low quality genotyping or those who were related to each other were excluded. In case of the latter, the patient with the latest (most recent) date of inclusion was excluded. Other reasons for exclusion during quality control were samples with likely sample contamination based on high degree of

relatedness with a large number of samples or when samples were >5 standard deviations from median for inbreeding coefficient, with a sex mismatch between genotype and phenotype[35]. For the PRS validation, further exclusions were made on the following basis: those with missing AAA or CVD phenotype information, controls that were aged 43 or under at the initial intake, controls that were lost to follow up, and cases that were under the age of 40 at intake. AAA cases were determined on 1) self-reported operations in AAA, 2) self-reported history of AAA, 3) inclusion in the SMART study because of an AAA, 4) infrarenal AAA measure >30mm or ratio of infrarenal:suprarenal aortic diameter > 1.5, or 5) finding of an AAA in the echo. This resulted in the inclusion of 663 cases and 3,105 controls in this validation dataset.

Bulk RNA sequencing from AAA patients: Abdominal aortic tissue was surgically resected during open repair for an abdominal aortic aneurysm. Samples were placed in a sterile field, partitioned into 0.5 cm x 0.5 cm pieces, placed in RNAlater (Qiagen GmbH, Hilden, Germany) and stored at room temperature between 1-3 days. The RNAlater solution was removed, and the tissue was stored at -80 °C. Tissues were cryopulverized using a CP02 instrument (Covaris), and an aliquot of the powdered tissue was used for isolation of total RNA using Trizol (Invitrogen) and RNeasy Mini Kit (Qiagen, GmbH, Hilden, Germany). Briefly, the RNA in the aqueous phase from the Trizol extraction was transferred to a new tube and mixed with an equal volume of 70% ethanol before processing on an RNeasy Mini kit column according to the manufacturer's instructions. RNA was eluted in 50ul of RNAse-free water. Concentrations were measured using the Qubit RNA Broad Range kit (Thermo Scientific, USA), followed by RNA integrity (RIN) evaluation by RNA TapeScreen on the Agilent 2200 TapeStation (Agilent Technologies, USA). RNA samples with a RIN > 7 were used in RNA sequencing analyses. Samples were prepared for sequencing using the Takara SMART-Seq v4 Ultra Low Input RNA Kit plus Nextera XT. Samples (n=126) underwent paired-end sequencing by MedGenome. FASTP[36] was used for adaptor trimming of the paired end reads. FASTQC was used for quality control. Reads were aligned to hg38 with annotation from GENCODE Human genome release 39 using STAR[37]. Samples were not included in downstream analysis if less than 70% STAR alignment, less than twenty thousand reads, or if the FASTQC GC content curve deviated significantly from a normal distribution centered around 50%. 15 abdominal aortic aneurysm RNA samples passed quality control. FeatureCounts[38] was used to aggregate a gene counts matrix. DESeq2[39] was used for variance stabilization transformation (VST) normalization. To determine if a gene was expressed

or not, a VST expression threshold of 6 was used. This cutoff was chosen based on a density plot of all transcript expression that yielded a bimodal distribution (**Supplementary Figure 17**). A cutoff of 6 effectively eliminated genes with no expression.

Conventional Mendelian Randomization

We performed conventional two-sample Mendelian randomization to estimate the total effect of each major lipoprotein-related trait on AAA. We constructed genetic instruments from independent ($r^2 < 0.001$, distance > 10,000kb) genetic variants associated with each major lipoprotein-related trait in the UK Biobank. After identifying the corresponding genetic variants in our GWAS of AAA and harmonizing the effect alleles, we performed MR using the *TwoSampleMR* package in R[40]. Our primary analysis used the inverse variance weighted method. In sensitivity analyses, we performed MR-Egger, weighted median, and weighted mode MR, which make different assumptions about the presence of pleiotropy[41].

MR-BMA

We performed a variable selection method in a multivariable Mendelian Randomization (MR) framework to prioritize the causal lipoprotein determinants of the outcomes. Multivariable MR extends the basic MR framework to include multiple exposures in one joint model, accounting for horizontal pleiotropy among exposures, which is particularly relevant when considering highly correlated traits like blood lipoprotein-related traits as exposures[42]. In order to rank and select the likely causal lipoprotein risk factors for AAA, we employed an extension of multivariable MR called Mendelian randomization Bayesian model averaging (MR-BMA), a Bayesian approach for prioritizing causal exposures in a two-sample multivariable MR setting[43]. MR-BMA performs variable selection by evaluating models with all possible combinations of lipoprotein-related traits as exposures and computing the posterior probability that the model contains the true causal risk factors. Unlike other univariate or multivariable MR methods, MR-BMA aims to identify true causal risk factors among correlated traits, rather than estimate the magnitude of effect. The marginal inclusion probability (level of evidential support for each exposure) is derived from the sum of all posterior probabilities of the models where the specific exposure was included. We removed influential variants based on the Cook's distance and outliers based on the q-statistic as previously recommended[44]. An empirical permutation procedure was performed to calculate p-

values. Briefly, the expected marginal inclusion probability distribution for each risk factor under the null hypothesis was generated by performing 1,000 permutations of the MR-BMA analysis, holding the SNP-risk factor associations constant and randomly permuting the SNP-outcome associations. The observed marginal inclusion probabilities for each risk factor were then compared to the expected distribution under the null, with p-values computed by $p_j = (r_j + 1)/(nperm + 1)$, where rj represents the rank of the observed marginal inclusion probability of a given risk factor (j) across all permutations (nperm = 1000). Adjustment for multiple testing was done using the Nyholt correction for correlated traits.

References

1. Willer, C.J., Li, Y. & Abecasis, G.R. METAL: fast and efficient meta-analysis of genomewide association scans. *Bioinformatics* **26**, 2190-2191 (2010).
2. Klarin, D., *et al.* Genetic Architecture of Abdominal Aortic Aneurysm in the Million Veteran Program. *Circulation* **142**, 1633-1646 (2020).
3. Roychowdhury, T., *et al.* Regulatory variants in TCF7L2 are associated with thoracic aortic aneurysm. *Am J Hum Genet* **108**, 1578-1589 (2021).
4. Weeks, E.M., *et al.* Leveraging polygenic enrichments of gene features to predict genes underlying complex traits and diseases. *Nat Genet* (2023).
5. Landrum, M.J., *et al.* ClinVar: improving access to variant interpretations and supporting evidence. *Nucleic Acids Res* **46**, D1062-D1067 (2018).
6. Renard, M., *et al.* Clinical Validity of Genes for Heritable Thoracic Aortic Aneurysm and Dissection. *J Am Coll Cardiol* **72**, 605-615 (2018).
7. Mountjoy, E., *et al.* An open approach to systematically prioritize causal variants and genes at all published human GWAS trait-associated loci. *Nat Genet* **53**, 1527-1533 (2021).
8. Pietzner, M., *et al.* Mapping the proteo-genomic convergence of human diseases. *Science* **374**, eabj1541 (2021).
9. Chen, H., *et al.* Author Correction: WWP2 regulates pathological cardiac fibrosis by modulating SMAD2 signaling. *Nat Commun* **10**, 4085 (2019).
10. Xie, C., *et al.* Yap1 protein regulates vascular smooth muscle cell phenotypic switch by interaction with myocardin. *J Biol Chem* **287**, 14598-14605 (2012).
11. Wang, L., *et al.* Yes-Associated Protein Inhibits Transcription of Myocardin and Attenuates Differentiation of Vascular Smooth Muscle Cell from Cardiovascular Progenitor Cell Lineage. *Stem Cells* **35**, 351-361 (2017).
12. Folsom, A.R., *et al.* Circulating Biomarkers and Abdominal Aortic Aneurysm Incidence: The Atherosclerosis Risk in Communities (ARIC) Study. *Circulation* **132**, 578-585 (2015).
13. Tang, W., *et al.* Lifetime Risk and Risk Factors for Abdominal Aortic Aneurysm in a 24-Year Prospective Study: The ARIC Study (Atherosclerosis Risk in Communities). *Arterioscler Thromb Vasc Biol* **36**, 2468-2477 (2016).
14. Marchini, J. & Howie, B. Genotype imputation for genome-wide association studies. *Nat Rev Genet* **11**, 499-511 (2010).
15. Sorensen, E., *et al.* Data Resource Profile: The Copenhagen Hospital Biobank (CHB). *Int J Epidemiol* **50**, 719-720e (2021).
16. Laursen, I.H., *et al.* Cohort profile: Copenhagen Hospital Biobank - Cardiovascular Disease Cohort (CHB-CVDC): Construction of a large-scale genetic cohort to facilitate a better understanding of heart diseases. *BMJ Open* **11**, e049709 (2021).
17. Hansen, T.F., *et al.* DBDS Genomic Cohort, a prospective and comprehensive resource for integrative and temporal analysis of genetic, environmental and lifestyle factors affecting health of blood donors. *BMJ Open* **9**, e028401 (2019).
18. Helgadottir, A., *et al.* Genetic variability in the absorption of dietary sterols affects the risk of coronary artery disease. *Eur Heart J* **41**, 2618-2628 (2020).
19. Zhou, W., *et al.* Efficiently controlling for case-control imbalance and sample relatedness in large-scale genetic association studies. *Nat Genet* **50**, 1335-1341 (2018).

20. Ho, D., Imai, K., King, G. & Stuart, E.A. MatchIt: Nonparametric Preprocessing for Parametric Causal Inference. *Journal of Statistical Software* **42**, 1 - 28 (2011).
21. Das, S.*, et al.* Next-generation genotype imputation service and methods. *Nat Genet* **48**, 1284-1287 (2016).
22. Kong, A.*, et al.* Detection of sharing by descent, long-range phasing and haplotype imputation. *Nat Genet* **40**, 1068-1075 (2008).
23. Gudbjartsson, D.F.*, et al.* Large-scale whole-genome sequencing of the Icelandic population. *Nat Genet* **47**, 435-444 (2015).
24. Jonsson, H.*, et al.* Whole genome characterization of sequence diversity of 15,220 Icelanders. *Sci Data* **4**, 170115 (2017).
25. Bulik-Sullivan, B.K.*, et al.* LD Score regression distinguishes confounding from polygenicity in genome-wide association studies. *Nat Genet* **47**, 291-295 (2015).
26. Stanaway, I.B.*, et al.* The eMERGE genotype set of 83,717 subjects imputed to ~40 million variants genome wide and association with the herpes zoster medical record phenotype. *Genet Epidemiol* **43**, 63-81 (2019).
27. Macrae, F.A., St John, D.J., Muir, E.P., Penfold, J.C. & Cuthbertson, A.M. Impact of a hospital-based register on the management of familial adenomatous polyposis. *Med J Aust* **151**, 552-557 (1989).
28. Krokstad, S.*, et al.* Cohort Profile: the HUNT Study, Norway. *Int J Epidemiol* **42**, 968-977 (2013).
29. Denny, J.C.*, et al.* Systematic comparison of phenome-wide association study of electronic medical record data and genome-wide association study data. *Nat Biotechnol* **31**, 1102-1110 (2013).
30. Loh, P.R.*, et al.* Reference-based phasing using the Haplotype Reference Consortium panel. *Nat Genet* **48**, 1443-1448 (2016).
31. Peypoch, O.*, et al.* The TAGA Study: A Study of Factors Determining Aortic Diameter in Families at High Risk of Abdominal Aortic Aneurysm Reveal Two New Candidate Genes. *J Clin Med* **9**(2020).
32. Vazquez-Santiago, M.*, et al.* Platelet count and plateletcrit are associated with an increased risk of venous thrombosis in females. Results from the RETROVE study. *Thromb Res* **157**, 162-164 (2017).
33. Bown, M.J.*, et al.* Abdominal aortic aneurysm is associated with a variant in low-density lipoprotein receptor-related protein 1. *Am J Hum Genet* **89**, 619-627 (2011).
34. Simons, P.C., Algra, A., van de Laak, M.F., Grobbee, D.E. & van der Graaf, Y. Second manifestations of ARTerial disease (SMART) study: rationale and design. *Eur J Epidemiol* **15**, 773-781 (1999).
35. Groenland, E.H.*, et al.* Genetic variants associated with low-density lipoprotein cholesterol and systolic blood pressure and the risk of recurrent cardiovascular disease in patients with established vascular disease. *Atherosclerosis* **350**, 102-108 (2022).
36. Chen, S., Zhou, Y., Chen, Y. & Gu, J. fastp: an ultra-fast all-in-one FASTQ preprocessor. *Bioinformatics* **34**, i884-i890 (2018).
37. Dobin, A.*, et al.* STAR: ultrafast universal RNA-seq aligner. *Bioinformatics* **29**, 15-21 (2013).
38. Liao, Y., Smyth, G.K. & Shi, W. featureCounts: an efficient general purpose program for assigning sequence reads to genomic features. *Bioinformatics* **30**, 923-930 (2014).

39. Love, M.I., Huber, W. & Anders, S. Moderated estimation of fold change and dispersion for RNA-seq data with DESeq2. *Genome Biol* **15**, 550 (2014).
40. Hemani, G.*, et al.* The MR-Base platform supports systematic causal inference across the human phenome. *Elife* **7**(2018).
41. Davies, N.M., Holmes, M.V. & Davey Smith, G. Reading Mendelian randomisation studies: a guide, glossary, and checklist for clinicians. *BMJ* **362**, k601 (2018).
42. Burgess, S. & Thompson, S.G. Multivariable Mendelian randomization: the use of pleiotropic genetic variants to estimate causal effects. *Am J Epidemiol* **181**, 251-260 (2015).
43. Zuber, V., Colijn, J.M., Klaver, C. & Burgess, S. Selecting likely causal risk factors from high-throughput experiments using multivariable Mendelian randomization. *Nat Commun* **11**, 29 (2020).
44. Zuber, V.*, et al.* High-throughput multivariable Mendelian randomization analysis prioritizes apolipoprotein B as key lipid risk factor for coronary artery disease. *Int J Epidemiol* **50**, 893-901 (2021).

Cohort acknowledgements

ARIC: The Atherosclerosis Risk in Communities (ARIC) Study has been funded in whole or in part with Federal funds from the National Heart, Lung, and Blood Institute, National Institutes of Health, Department of Health and Human Services, under Contract nos. (HHSN268201700001I, HHSN268201700002I, HHSN268201700003I, HHSN268201700004I, HHSN268201700005I). The authors thank the staff and participants of the ARIC study for their important contributions. Funding was also supported by R01HL103695, R01HL155209, R01HL087641, R01HL059367 and R01HL086694; National Human Genome Research Institute contract U01HG004402; and National Institutes of Health contract HHSN268200625226C. Infrastructure was partly supported by Grant Number UL1RR025005, a component of the National Institutes of Health and NIH Roadmap for Medical Research. Jack W. Pattee was supported by NIH T32GM108557.

eMERGE: eMERGE Network (Phase III): This phase of the eMERGE Network was initiated and funded by the NHGRI through the following grants: U01HG8657 (Group Health Cooperative/University of Washington); U01HG8685 (Brigham and Women's Hospital); U01HG8672 (Vanderbilt University Medical Center); U01HG8666 (Cincinnati Children's Hospital Medical Center); U01HG6379 (Mayo Clinic); U01HG8679 (Geisinger Clinic); U01HG8680 (Columbia University Health Sciences); U01HG8684 (Children's Hospital of Philadelphia); U01HG8673 (Northwestern University); U01HG8701 (Vanderbilt University Medical Center serving as the Coordinating Center); U01HG8676 (Partners Healthcare/Broad Institute); and U01HG8664 (Baylor College of Medicine). We would also like to acknowledge the following eMERGE members who contributed to the eMERGE data for this manuscript: D. Crosslin, J. Denny, M. Palmer, SA. Pendergrass, and I. Stanaway.

HUNT: The Trøndelag Health Study (The HUNT Study) is a collaboration between HUNT Research Center (Faculty of Medicine and Health Sciences, NTNU, Norwegian University of Science and Technology), Trøndelag County Council, Central Norway Regional Health Authority, and the Norwegian Institute of Public Health. The genotyping in HUNT was financed by the National Institutes of Health; University of Michigan; the Research Council of Norway; the Liaison Committee for Education, Research and Innovation in Central Norway; and the Joint Research Committee between St Olav's hospital and the Faculty of Medicine and Health Sciences, NTNU.

TABS: TABS is funded by grant PID2019-109844RB-I00 from the Spanish Ministry of Science and Innovation. The genotyping service was carried out at CEGEN-PRB3-ISCIII and supported by grant PT17/0019, of the PE I+D+i 2013-2016, funded by ISCIII and ERDF.

// Consortium authors

DiscovEHR

Regeneron personnel:
Goncalo Abecasis, Aris Baras, Michael Cantor, Giovanni Coppola, Aris Economides, Luca A. Lotta, John D. Overton, Jeffrey G. Reid, Alan Shuldiner, Andrew Deubler, Katia Karalis, Christina Beechert, Caitlin Forsythe, Erin D. Fuller, Zhenhua Gu, Michael Lattari, Alexander Lopez, Thomas D. Schleicher, Maria Sotiropoulos Padilla, Karina Toledo, Louis Widom, Sarah E. Wolf, Manasi Pradhan, Kia Manoochehri, Ricardo H. Ulloa, Xiaodong Bai, Suganthi Balasubramanian, Leland Barnard, Andrew Blumenfeld, Gisu Eom, Lukas Habegger, Alicia Hawes, Shareef Khalid, Evan K. Maxwell, William Salerno, Jeffrey C. Staples, Ashish Yadav, Dadong Li, Marcus B. Jones, Lyndon J. Mitnaul, Jason Mighty, Andrew Deubler, Katia Karalis, Katherine Siminovitch

Geisinger personnel:
Lance J. Adams, Jackie Blank, Dale Bodian, Derek Boris, Adam Buchanan, David J. Carey, Ryan D. Colonie, F. Daniel Davis, Dustin N. Hartzel, Melissa Kelly, H. Lester Kirchner, Joseph B. Leader, David H. Ledbetter, Ph.D., J. Neil Manus, Christa L. Martin, Raghu P. Metpally, Michelle Meyer, Tooraj Mirshahi, Matthew Oetjens, Thomas Nate Person, Christopher Still, Natasha Strande, Amy Sturm, Jen Wagner, Marc Williams

Regeneron Genetics Center

RGC Management and Leadership Team
Goncalo Abecasis, D.Phil. , Aris Baras, M.D. , Michael Cantor, M.D. , Giovanni Coppola, M.D. , Andrew Deubler , Aris Economides, Ph.D. , Katia Karalis, Ph.D. , Luca A. Lotta, M.D., Ph.D. , John D. Overton, Ph.D. , Jeffrey G. Reid, Ph.D. , Katherine Siminovitch, M.D. , Alan Shuldiner, M.D

Sequencing and Lab Operations
Christina Beechert , Caitlin Forsythe, M.S. , Erin D. Fuller , Zhenhua Gu, M.S. , Michael Lattari , Alexander Lopez, M.S., John D. Overton, Ph.D. , Maria Sotiropoulos Padilla, M.S. , Manasi Pradhan, M.S. , Kia Manoochehri, B.S. , Thomas D. Schleicher, M.S. , Louis Widom , Sarah E. Wolf, M.S. , Ricardo H. Ulloa, B.S.

Clinical Informatics
Amelia Averitt, Ph.D. , Nilanjana Banerjee, Ph.D. , Michael Cantor, M.D. , Dadong Li, Ph.D. , Sameer Malhotra, M.D. , Deepika Sharma, MHI , Jeffrey Staples , Ph.D.

Genome Informatics
Xiaodong Bai, Ph.D. , Suganthi Balasubramanian, Ph.D. , Suying Bao, Ph.D. , Boris Boutkov, Ph.D. , Siying Chen, Ph.D. , Gisu Eom, B.S. , Lukas Habegger, Ph.D. , Alicia Hawes, B.S. , Shareef Khalid , Olga Krasheninina, M.S. , Rouel Lanche, B.S. , Adam J. Mansfield, B.A. , Evan

K. Maxwell, Ph.D. , George Mitra, B.A. , Mona Nafde, M.S. , Sean O'Keeffe, Ph.D. , Max Orelus, B.B.A. , Razvan Panea, Ph.D. , Tommy Polanco, B.A. , Ayesha Rasool, M.S. , Jeffrey G. Reid, Ph.D. , William Salerno, Ph.D. , Jeffrey C. Staples, Ph.D. , Kathie Sun, Ph.D. , Jiwen Xin, Ph.D.

Analytical Genomics and Data Science
Goncalo Abecasis, D.Phil. , Joshua Backman, Ph.D. , Amy Damask, Ph.D. , Lee Dobbyn, Ph.D. , Manuel Allen Revez Ferreira, Ph.D. , Arkopravo Ghosh, M.S. , Christopher Gillies, Ph.D. , Lauren Gurski, B.S. , Eric Jorgenson, Ph.D. , Hyun Min Kang, Ph.D. , Michael Kessler, Ph.D. , Jack Kosmicki, Ph.D. , Alexander Li , Ph.D. , Nan Lin, Ph.D. , Daren Liu, M.S. , Adam Locke, Ph.D. , Jonathan Marchini, Ph.D. , Anthony Marcketta, M.S. , Joelle Mbatchou, Ph.D. , Arden Moscati, Ph.D. , Charles Paulding, Ph.D. , Carlo Sidore, Ph.D. , Eli Stahl, Ph.D. , Kyoko Watanabe, Ph.D. , Bin Ye, Ph.D. , Blair Zhang, Ph.D. , Andrey Ziyatdinov, Ph.D.

Therapeutic Area Genetics
Ariane Ayer, B.S. , Aysegul Guvenek, Ph.D. , George Hindy, Ph.D. , Giovanni Coppola, M.D. , Jan Freudenberg, M.D. , Jonas Bovijn M.D. , Julie Horowitz, Ph.D. , Katherine Siminovitch, M.D. , Jonas B. Nielsen, MD, PhD, Kavita Praveen, Ph.D. , Luca A. Lotta, M.D. , Manav Kapoor, Ph.D. , Mary Haas, Ph.D. , Moeen Riaz , Ph.D. , Niek Verweij, Ph.D. , Olukayode Sosina, Ph.D. , Parsa Akbari, Ph.D. , Priyanka Nakka, Ph.D. , Sahar Gelfman, Ph.D. , Sujit Gokhale, B.E. , Tanima De, Ph.D. , Veera Rajagopal, Ph.D. , Alan Shuldiner, M.D. , Bin Ye, Ph.D. , Gannie Tzoneva, Ph.D. , Juan Rodriguez-Flores, Ph.D.

RGC Biology
Shek Man Chim, Ph.D. , Valerio Donato, Ph.D. , Aris Economides, Ph.D. , Daniel Fernandez, M.S. , Giusy Della Gatta, Ph.D. , Alessandro Di Gioia, Ph.D. , Kristen Howell, M.S. , Katia Karalis, Ph.D. , Lori Khrimian, Ph.D. , Minhee Kim, Ph.D. , Hector Martinez , Lawrence Miloscio, B.S. , Sheilyn Nunez, B.S. , Elias Pavlopoulos, Ph.D. , Trikaldarshi Persaud, B.S.

Research Program Management & Strategic Initiatives
Esteban Chen, M.S. , Marcus B. Jones, Ph.D. , Michelle G. LeBlanc, Ph.D. , Jason Mighty, Ph.D. , Lyndon J. Mitnaul, Ph.D. , Nirupama Nishtala, Ph.D. , Nadia Rana, Ph.D.

UK Aneurysm Growth Study

Sara Baker, Jamie Barwell, Marcus Brooks, Neil Browning, Ian Chetter, Sohail Choksy, Alun Davies, Mark Dayer, Jonothan Earnshaw, Louis Fligelstone, Mark Gannon, Eric Grocott, Paul Hayes, Chris Imray, Nilesh Samani, Tim Lees, Gabor Libertiny, Charles McCollum, Colin Nice, Rajiv Pathak, Arun Pherwani, Lynda Pike, John Quarmby, Thomas Rix, Rob Sayers, Cliff Shearman, Vince Smyth, Mike Sweeting, Tim Sykes, William Tennant, John Thompson, Rao Vallabhaneni, Syed Yusuf, Frank Dudbridge

DBDS Genomic Consortium

Steffen Andersen[1], Karina Banasik[2], Søren Brunak[2], Kristoffer Burgdorf[3], Maria Didriksen[3], Khoa Manh Dinh[4], Christian Erikstrup[4], Daniel Gudbjartsson[5], Thomas Folkmann Hansen[6], Henrik Hjalgrim[7], Gregor Jemec[8], Poul Jennum[9], Pär Ingemar Johansson[3], Margit Anita Hørup Larsen[3], Susan Mikkelsen[4], Kasper Rene Nielsen[10], Mette Nyegaard[11], Sisse Rye Ostrowski[3], Ole Birger Pedersen[12], Kari Stefansson[5], Hreinn Stefánsson[5], Susanne Sækmose[12], Erik Sørensen[3], Unnur Þorsteinsdóttir[5], Mie Topholm Brun[13], Henrik Ullum[14], Thomas Werge[15]

[1] Department of Finance, Copenhagen Business School, Copenhagen, Denmark
[2] Novo Nordisk Foundation Center for Protein Research, Faculty of Health and Medical Sciences, University of Copenhagen, Copenhagen, Denmark
[3] Department of Clinical Immunology, Copenhagen University Hospital – Rigshospitalet, Copenhagen, Denmark
[4] Department of Clinical Immunology, Aarhus University Hospital, Aarhus
[5] deCODE Genetics, Reykjavik, Iceland
[6] Danish Headache Center, Department of Neurology, Copenhagen University Hospital, Rigshospitalet – Glostrup
[7] Department of Epidemiology Research, Statens Serum Institut, Centre for Cancer Research, Danish Cancer Society, Copenhagen, Denmark
[8] Department of Clinical Medicine, Sealand University hospital – Roskilde, Roskilde, Denmark
[9] Department of clinical neurophysiology, University of Copenhagen, Copenhagen, Denmark
[10] Department of Clinical Immunology, Aalborg University Hospital, Aalborg, Denmark
[11] Department of Biomedicine, Aarhus University, Aarhus, Denmark
[12] Department of Clinical Immunology, Zealand University Hospital – Køge, Køge, Denmark
[13] Department of Clinical Immunology, Odense University Hospital, Odense, Denmark
[14] Statens Serum Institute, Copenhagen, Denmark
[15] Institute of Biological Psychiatry Mental Health Centre, Sct. Hans, Copenhagen University Hospital – Roskilde, Roskilde, Denmark

VA Million Veteran Program

Core Acknowledgement for Publications
February 2023

MVP Program Office

- Sumitra Muralidhar, Ph.D., Program Director
  US Department of Veterans Affairs, 810 Vermont Avenue NW, Washington, DC 20420
- Jennifer Moser, Ph.D., Associate Director, Scientific Programs
  US Department of Veterans Affairs, 810 Vermont Avenue NW, Washington, DC 20420
- Jennifer E. Deen, B.S., Associate Director, Cohort & Public Relations
  US Department of Veterans Affairs, 810 Vermont Avenue NW, Washington, DC 20420

MVP Executive Committee

- Co-Chair: Philip S. Tsao, Ph.D.

VA Palo Alto Health Care System, 3801 Miranda Avenue, Palo Alto, CA 94304
- Co-Chair: Sumitra Muralidhar, Ph.D.
  US Department of Veterans Affairs, 810 Vermont Avenue NW, Washington, DC 20420
- J. Michael Gaziano, M.D., M.P.H.
  VA Boston Healthcare System, 150 S. Huntington Avenue, Boston, MA 02130
- Elizabeth Hauser, Ph.D.
  Durham VA Medical Center, 508 Fulton Street, Durham, NC 27705
- Amy Kilbourne, Ph.D., M.P.H.
  VA HSR&D, 2215 Fuller Road, Ann Arbor, MI 48105
- Shiuh-Wen Luoh, M.D., Ph.D.
  VA Portland Health Care System, 3710 SW US Veterans Hospital Rd, Portland, OR 97239
- Michael Matheny, M.D., M.S., M.P.H.
  VA Tennessee Valley Healthcare System, 1310 24th Ave. South, Nashville, TN 37212
- Dave Oslin, M.D.
  Philadelphia VA Medical Center, 3900 Woodland Avenue, Philadelphia, PA 19104

MVP Co-Principal Investigators

- J. Michael Gaziano, M.D., M.P.H.
  VA Boston Healthcare System, 150 S. Huntington Avenue, Boston, MA 02130
- Philip S. Tsao, Ph.D.
  VA Palo Alto Health Care System, 3801 Miranda Avenue, Palo Alto, CA 94304

MVP Core Operations

- Lori Churby, B.S., Director, MVP Regulatory Affairs
  VA Palo Alto Health Care System, 3801 Miranda Avenue, Palo Alto, CA 94304
- Stacey B. Whitbourne, Ph.D., Director, MVP Cohort Management
  VA Boston Healthcare System, 150 S. Huntington Avenue, Boston, MA 02130
- Jessica V. Brewer, M.P.H., Director, MVP Recruitment & Enrollment
  VA Boston Healthcare System, 150 S. Huntington Avenue, Boston, MA 02130
- Shahpoor (Alex) Shayan, M.S., Director, MVP Recruitment and Enrollment Informatics
  VA Boston Healthcare System, 150 S. Huntington Avenue, Boston, MA 02130
- Luis E. Selva, Ph.D., Executive Director, MVP Biorepositories
  VA Boston Healthcare System, 150 S. Huntington Avenue, Boston, MA 02130
- Saiju Pyarajan Ph.D., Director, Data and Computational Sciences
  VA Boston Healthcare System, 150 S. Huntington Avenue, Boston, MA 02130
- Kelly Cho, M.P.H, Ph.D., Director, MVP Phenomics Data Core
  VA Boston Healthcare System, 150 S. Huntington Avenue, Boston, MA 02130
- Scott L. DuVall, Ph.D., Director, VA Informatics and Computing Infrastructure (VINCI)
  VA Salt Lake City Health Care System, 500 Foothill Drive, Salt Lake City, UT 84148
- Mary T. Brophy M.D., M.P.H., Director, VA Central Biorepository
  VA Boston Healthcare System, 150 S. Huntington Avenue, Boston, MA 02130
- MVP Coordinating Centers
  - MVP Coordinating Center, Boston - J. Michael Gaziano, M.D., M.P.H.

VA Boston Healthcare System, 150 S. Huntington Avenue, Boston, MA 02130

- MVP Coordinating Center, Palo Alto – Philip S. Tsao, Ph.D.
  VA Palo Alto Health Care System, 3801 Miranda Avenue, Palo Alto, CA 94304
- MVP Information Center, Canandaigua – Brady Stephens, M.S.
  Canandaigua VA Medical Center, 400 Fort Hill Avenue, Canandaigua, NY 14424
- Cooperative Studies Program Clinical Research Pharmacy Coordinating Center, Albuquerque – Todd Connor, Pharm.D.; Dean P. Argyres, B.S., M.S.
  New Mexico VA Health Care System, 1501 San Pedro Drive SE, Albuquerque, NM 87108

MVP Publications and Presentations Committee

- Co-Chair: Tim Assimes, M.D.
  VA Palo Alto Health Care System, 3801 Miranda Avenue, Palo Alto, CA 94304
- Co-Chair: Adriana Hung, M.D.
  VA Tennessee Valley Healthcare System, 1310 24th Ave. South, Nashville, TN 37212
- Co-Chair: Henry Kranzler, M.D.
  Philadelphia VA Medical Center, 3900 Woodland Avenue, Philadelphia, PA 19104

MVP Local Site Investigators

- Samuel Aguayo, M.D., Phoenix VA Health Care System
  650 E. Indian School Road, Phoenix, AZ 85012
- Sunil Ahuja, M.D., South Texas Veterans Health Care System
  7400 Merton Minter Boulevard, San Antonio, TX 78229
- Kathrina Alexander, M.D., Veterans Health Care System of the Ozarks
  1100 North College Avenue, Fayetteville, AR 72703
- Xiao M. Androulakis, M.D., Columbia VA Health Care System
  6439 Garners Ferry Road, Columbia, SC 29209
- Prakash Balasubramanian, M.D., William S. Middleton Memorial Veterans Hospital
  2500 Overlook Terrace, Madison, WI 53705
- Zuhair Ballas, M.D., Iowa City VA Health Care System
  601 Highway 6 West, Iowa City, IA 52246-2208
- Jean Beckham, Ph.D., Durham VA Medical Center
  508 Fulton Street, Durham, NC 27705
- Sujata Bhushan, M.D., VA North Texas Health Care System
  4500 S. Lancaster Road, Dallas, TX 75216
- Edward Boyko, M.D., VA Puget Sound Health Care System
  1660 S. Columbian Way, Seattle, WA 98108-1597
- David Cohen, M.D., Portland VA Medical Center
- 3710 SW U.S. Veterans Hospital Road, Portland, OR 97239
- Dellitalia, M.D., Birmingham VA Medical Center
  700 S. 19th Street, Birmingham AL 35233
- L. Christine Faulk, M.D., Robert J. Dole VA Medical Center
  5500 East Kellogg Drive, Wichita, KS 67218-1607
- Joseph Fayad, M.D., VA Southern Nevada Healthcare System

6900 North Pecos Road, North Las Vegas, NV 89086
- Daryl Fujii, Ph.D., VA Pacific Islands Health Care System
459 Patterson Rd, Honolulu, HI 96819
- Saib Gappy, M.D., John D. Dingell VA Medical Center
4646 John R Street, Detroit, MI 48201
- Frank Gesek, Ph.D., White River Junction VA Medical Center
163 Veterans Drive, White River Junction, VT 05009
- Jennifer Greco, M.D., Sioux Falls VA Health Care System
2501 W 22nd Street, Sioux Falls, SD 57105
- Michael Godschalk, M.D., Richmond VA Medical Center
1201 Broad Rock Blvd., Richmond, VA 23249
- Todd W. Gress, M.D., Ph.D., Hershel "Woody" Williams VA Medical Center
1540 Spring Valley Drive, Huntington, WV 25704
- Samir Gupta, M.D., M.S.C.S., VA San Diego Healthcare System
3350 La Jolla Village Drive, San Diego, CA 92161
- Salvador Gutierrez, M.D., Edward Hines, Jr. VA Medical Center
5000 South 5th Avenue, Hines, IL 60141
- John Harley, M.D., Ph.D., Cincinnati VA Medical Center
3200 Vine Street, Cincinnati, OH 45220
- Kimberly Hammer, Ph.D., Fargo VA Health Care System
2101 N. Elm, Fargo, ND 58102
- Mark Hamner, M.D., Ralph H. Johnson VA Medical Center
109 Bee Street, Mental Health Research, Charleston, SC 29401
- Adriana Hung, M.D., M.P.H., VA Tennessee Valley Healthcare System
1310 24th Avenue, South Nashville, TN 37212
- Robin Hurley, M.D., W.G. (Bill) Hefner VA Medical Center
1601 Brenner Ave, Salisbury, NC 28144
- Pran Iruvanti, D.O., Ph.D., Hampton VA Medical Center
100 Emancipation Drive, Hampton, VA 23667
- Frank Jacono, M.D., VA Northeast Ohio Healthcare System
10701 East Boulevard, Cleveland, OH 44106
- Darshana Jhala, M.D., Philadelphia VA Medical Center
3900 Woodland Avenue, Philadelphia, PA 19104
- Scott Kinlay, M.B.B.S., Ph.D., VA Boston Healthcare System
150 S. Huntington Avenue, Boston, MA 02130
- Jon Klein, M.D., Ph.D., Louisville VA Medical Center
800 Zorn Avenue, Louisville, KY 40206
- Michael Landry, Ph.D., Southeast Louisiana Veterans Health Care System
2400 Canal Street, New Orleans, LA 70119
- Peter Liang, M.D., M.P.H., VA New York Harbor Healthcare System
423 East 23rd Street, New York, NY 10010
- Suthat Liangpunsakul, M.D., M.P.H., Richard Roudebush VA Medical Center
1481 West 10th Street, Indianapolis, IN 46202
- Jack Lichy, M.D., Ph.D., Washington DC VA Medical Center
50 Irving St, Washington, D. C. 20422
- C. Scott Mahan, M.D., Charles George VA Medical Center

1100 Tunnel Road, Asheville, NC 28805

- Ronnie Marrache, M.D., VA Maine Healthcare System
  1 VA Center, Augusta, ME 04330
- Stephen Mastorides, M.D., James A. Haley Veterans' Hospital
  13000 Bruce B. Downs Blvd, Tampa, FL 33612
- Elisabeth Mates M.D., Ph.D., VA Sierra Nevada Health Care System
  975 Kirman Avenue, Reno, NV 89502
- Kristin Mattocks, Ph.D., M.P.H., Central Western Massachusetts Healthcare System
  421 North Main Street, Leeds, MA 01053
- Paul Meyer, M.D., Ph.D., Southern Arizona VA Health Care System
  3601 S 6th Avenue, Tucson, AZ 85723
- Jonathan Moorman, M.D., Ph.D., James H. Quillen VA Medical Center
  Corner of Lamont & Veterans Way, Mountain Home, TN 37684
- Timothy Morgan, M.D., VA Long Beach Healthcare System
  5901 East 7th Street Long Beach, CA 90822
- Maureen Murdoch, M.D., M.P.H., Minneapolis VA Health Care System
  One Veterans Drive, Minneapolis, MN 55417
- James Norton, Ph.D., VA Health Care Upstate New York
  113 Holland Avenue, Albany, NY 12208
- Olaoluwa Okusaga, M.D., Michael E. DeBakey VA Medical Center
  2002 Holcombe Blvd, Houston, TX 77030
- Kris Ann Oursler, M.D., Salem VA Medical Center
  1970 Roanoke Blvd, Salem, VA 24153
- Ana Palacio, M.D., M.P.H., Miami VA Health Care System
  1201 NW 16th Street, 11 GRC, Miami FL 33125
- Samuel Poon, M.D., Manchester VA Medical Center
  718 Smyth Road, Manchester, NH 03104
- Emily Potter, Pharm.D., VA Eastern Kansas Health Care System
  4101 S 4th Street Trafficway, Leavenworth, KS 66048
- Michael Rauchman, M.D., St. Louis VA Health Care System
  915 North Grand Blvd, St. Louis, MO 63106
- Richard Servatius, Ph.D., Syracuse VA Medical Center
  800 Irving Avenue, Syracuse, NY 13210
- Satish Sharma, M.D., Providence VA Medical Center
  830 Chalkstone Avenue, Providence, RI 02908
- River Smith, Ph.D., Eastern Oklahoma VA Health Care System
  1011 Honor Heights Drive, Muskogee, OK 74401
- Peruvemba Sriram, M.D., N. FL/S. GA Veterans Health System
  1601 SW Archer Road, Gainesville, FL 32608
- Patrick Strollo, Jr., M.D., VA Pittsburgh Health Care System
  University Drive, Pittsburgh, PA 15240
- Neeraj Tandon, M.D., Overton Brooks VA Medical Center
  510 East Stoner Ave, Shreveport, LA 71101
- Philip Tsao, Ph.D., VA Palo Alto Health Care System
  3801 Miranda Avenue, Palo Alto, CA 94304-1290
- Gerardo Villareal, M.D., New Mexico VA Health Care System

1501 San Pedro Drive, S.E. Albuquerque, NM 87108

- Agnes Wallbom, M.D., M.S., VA Greater Los Angeles Health Care System
  11301 Wilshire Blvd, Los Angeles, CA 90073
- Jessica Walsh, M.D., VA Salt Lake City Health Care System
  500 Foothill Drive, Salt Lake City, UT 84148
- John Wells, Ph.D., Edith Nourse Rogers Memorial Veterans Hospital
  200 Springs Road, Bedford, MA 01730
- Jeffrey Whittle, M.D., M.P.H., Clement J. Zablocki VA Medical Center
  5000 West National Avenue, Milwaukee, WI 53295
- Mary Whooley, M.D., San Francisco VA Health Care System
  4150 Clement Street, San Francisco, CA 94121
- Allison E. Williams, N.D., Ph.D., R.N, Bay Pines VA Healthcare System
  10,000 Bay Pines Blvd Bay Pines, FL 33744
- Peter Wilson, M.D., Atlanta VA Medical Center
  1670 Clairmont Road, Decatur, GA 30033
- Junzhe Xu, M.D., VA Western New York Healthcare System
  3495 Bailey Avenue, Buffalo, NY 14215-1199
- Shing Shing Yeh, Ph.D., M.D., Northport VA Medical Center
  79 Middleville Road, Northport, NY 11768
