# Supplementary material for: Genome-wide association meta-analysis identifies risk loci for abdominal aortic aneurysm and highlights PCSK9 as a therapeutic target
Source: Nat Genet. 2023 Oct 16;55(11):1831–42. doi: 10.1038/s41588-023-01510-y (PMC10632148; doi:10.1038/s41588-023-01510-y)
Supplement: Supplementary file 2 — Reporting Summary [file 41588_2023_1510_MOESM2_ESM.pdf]

nature portfolio

Corresponding author(s): Scott M Damrauer
Cristen J Willer
Tanmoy Roychowdhury

Last updated by author(s): Aug 11, 2023

Reporting Summary

Nature Portfolio wishes to improve the reproducibility of the work that we publish. This form provides structure for consistency and transparency in reporting. For further information on Nature Portfolio policies, see our Editorial Policies and the Editorial Policy Checklist.

Statistics

For all statistical analyses, confirm that the following items are present in the figure legend, table legend, main text, or Methods section.

n/a	Confirmed	
☐	☒	The exact sample size (*n*) for each experimental group/condition, given as a discrete number and unit of measurement
☐	☒	A statement on whether measurements were taken from distinct samples or whether the same sample was measured repeatedly
☐	☒	The statistical test(s) used AND whether they are one- or two-sided<br>*Only common tests should be described solely by name; describe more complex techniques in the Methods section.*
☐	☒	A description of all covariates tested
☐	☒	A description of any assumptions or corrections, such as tests of normality and adjustment for multiple comparisons
☐	☒	A full description of the statistical parameters including central tendency (e.g. means) or other basic estimates (e.g. regression coefficient) AND variation (e.g. standard deviation) or associated estimates of uncertainty (e.g. confidence intervals)
☐	☒	For null hypothesis testing, the test statistic (e.g. *F*, *t*, *r*) with confidence intervals, effect sizes, degrees of freedom and *P* value noted<br>*Give P values as exact values whenever suitable.*
☐	☒	For Bayesian analysis, information on the choice of priors and Markov chain Monte Carlo settings
☐	☒	For hierarchical and complex designs, identification of the appropriate level for tests and full reporting of outcomes
☐	☒	Estimates of effect sizes (e.g. Cohen's *d*, Pearson's *r*), indicating how they were calculated

Our web collection on statistics for biologists contains articles on many of the points above.

Software and code

Policy information about availability of computer code

Data collection	No software was used for data collection.
Data analysis	Software tools used for discovery cohort GWAS are outlined in the Supplementary Note. Publicly available software tools were used to perform meta-analysis and downstream analysis. These tools include METAL 2011-03-25 (http://csg.sph.umich.edu/abecasis/Metal/), PRS-cs v1.0.0-Apr 11, 2020 (https://github.com/getian107/PRScs), GCTA 1.92.1 (https://yanglab.westlake.edu.cn/software/gcta/), DEPICT version 1 rel194 (https://github.com/perslab/depict), LDSC v1.0.0 (https://github.com/bulik/ldsc), RolyPoly (https://github.com/dcalderon/rolypoly), VEP (https://useast.ensembl.org/info/docs/tools/vep/), PoPS v0.2 (https://github.com/FinucaneLab/pops), Coloc v3.2.1 (https://cran.r-project.org/web/packages/coloc/), MetaXcan v0.7.5 (https://github.com/hakyimlab/MetaXcan), Gephi (https://github.com/gephi/gephi), MR-BMA v2021-10-05 (https://github.com/verena-zuber/demo_AMD), TwoSampleMR v0.5.6 (https://mrcieu.github.io/TwoSampleMR/), HyPrColoc v2021-07-23 (https://github.com/jrs95/hyprcoloc).

For manuscripts utilizing custom algorithms or software that are central to the research but not yet described in published literature, software must be made available to editors and reviewers. We strongly encourage code deposition in a community repository (e.g. GitHub). See the Nature Portfolio guidelines for submitting code & software for further information.

Data

Policy information about availability of data

All manuscripts must include a data availability statement. This statement should provide the following information, where applicable:

- Accession codes, unique identifiers, or web links for publicly available datasets
- A description of any restrictions on data availability
- For clinical datasets or third party data, please ensure that the statement adheres to our policy

Meta-analysis summary statistics and PRS weights are available here: https://csg.sph.umich.edu/willer/public/AAAgen2023/. Unpublished mouse transcriptome data is available at GEO (GSE197748). Due to stipulations of the IRB, abdominal aortic aneurysm bulk RNA-seq data is only available directly from the authors (isurakka@med.umich.edu) and will be provided to qualified investigators with appropriate IRB approval and materials transfer agreement.

Human research participants

Policy information about studies involving human research participants and Sex and Gender in Research.

Reporting on sex and gender	The results are not sex-stratified. As majority of AAA cases occurs in the male sex, dataset primarily consists of male sex. GWAS analysis in discovery cohorts incorporated sex as a covariate.
Population characteristics	The meta-analysis incorporates data from individuals of European and African ancestry. Following covariates were used for discovery cohort GWAS: ARIC (Age, Sex, Pack years of smoking, PCs 1-5); CHB-CVDC+DBDS (Birth year, Sex, PCs 1-10); CHIP +MGI (Birth year, Sex, Array version, PCs 1-4); deCODE (Age, Age2, Sex, County of origin, Some additional); DiscovEHR (Age, Age2, Sex, AgexSex, Age2xSex, PCs1-10); eMERGE (Age, Sex, Clinical site, PCs1-5); HUNT (Birth year, Sex, Batch, PCs 1-4); Mayo VDB (Study enrolment age, Sex, Genotyping platform, PCs 1-5); MVP (Age, Sex, PCs 1-5); NZ (NA); PMBB (Age at recruitment, Sex, Genetic determined ancestry, PCs 1-10); TABS (Birth year, Sex, Batch, PCs 1-4); UKAGS+VIVA (Birth year, Sex, Batch, PCs 1-4); UKBB (Age matching for controls, Sex). These information are also listed in Supplementary Table 1.
Recruitment	Outlined in Supplementary Methods relevant to discovery cohorts.
Ethics oversight	Outlined in Supplementary Methods relevant to discovery cohorts.

Note that full information on the approval of the study protocol must also be provided in the manuscript.

Field-specific reporting

Please select the one below that is the best fit for your research. If you are not sure, read the appropriate sections before making your selection.

☒ Life sciences ☐ Behavioural & social sciences ☐ Ecological, evolutionary & environmental sciences

For a reference copy of the document with all sections, see nature.com/documents/nr-reporting-summary-flat.pdf

Life sciences study design

All studies must disclose on these points even when the disclosure is negative.

Sample size	Sample size was determined based on using all genetic data (European and African ancestry) available from 14 discovery cohorts. Participants were excluded if they failed to meet case or control definitions.
Data exclusions	Data were excluded if they did not pass our QC metrics.
Replication	External datasets lack power for replication. We demonstrated consistent effect size estimation (Supplementary Figure 7, Supplementary Table 2) using Finngen and PRS validation cohorts that were not incorporated in the meta-analysis ($P<0.05$ in 80/121 loci).
Randomization	Randomization is not relevant for this binary trait GWAS study which is a retrospective analysis of AAA cases and controls.
Blinding	Randomization is not relevant for this binary trait GWAS study which is a retrospective analysis of AAA cases and controls.

Reporting for specific materials, systems and methods

We require information from authors about some types of materials, experimental systems and methods used in many studies. Here, indicate whether each material, system or method listed is relevant to your study. If you are not sure if a list item applies to your research, read the appropriate section before selecting a response.

Materials & experimental systems

n/a	Involved in the study
☒	☐ Antibodies
☒	☐ Eukaryotic cell lines
☒	☐ Palaeontology and archaeology
☐	☒ Animals and other organisms
☒	☐ Clinical data
☒	☐ Dual use research of concern

Methods

n/a	Involved in the study
☒	☐ ChIP-seq
☒	☐ Flow cytometry
☒	☐ MRI-based neuroimaging

Animals and other research organisms

Policy information about studies involving animals; ARRIVE guidelines recommended for reporting animal research, and Sex and Gender in Research

Laboratory animals	10 week-old male C57Bl/6 and PCSK9 (-/-) mice on a C57Bl/6 background were used in the current study. Animals were housed in the animal facility at the VA Palo Alto Health Care System which was temperature (68-72 F)- and humidity (70%)-controlled under a 12-h light/dark cycle.
Wild animals	No wild animals were used in this study.
Reporting on sex	Male mice were used in this study as the majority of AAA cases occurs in the male sex.
Field-collected samples	No field collected samples were used in this study.
Ethics oversight	All animal protocols were approved by the Administrative Panel on Laboratory Animal Care at Stanford University (http://labanimals.stanford.edu/) and the VA Palo Alto Health Care System Institutional Animal Care

Note that full information on the approval of the study protocol must also be provided in the manuscript.
